# Aerobiological and clinical study in the semidesertic area of the Southeastern of Spain

**DOI:** 10.3389/falgy.2024.1328940

**Published:** 2024-03-25

**Authors:** Juan José Zapata, Laura Martín-López, Laura Bosch, Jorge del Campo, Jerónimo Carnés

**Affiliations:** ^1^ Private Allergy Clinic, Dr. Juan José Zapata Yébenes, Almería, Spain; ^2^ R&D Allergy & Immunology Unit, LETI Pharma S.L.U., Madrid, Spain; ^3^ Biostatistics Specialist Freelance, Madrid, Spain

**Keywords:** aerobiology, weather influence, Oleaceae, temperature, pollen count, prevalence

## Abstract

Aerobiological studies constitute a relevant tool to predict the most influential parameters over the pollen seasons with significant clinical relevance in the allergic populations. The aim of this study was to describe the aerobiological behaviour of the most relevant allergenic sources in the semi-arid area of southeast of Spain (Almería) and to investigate the correlation with meteorological factors and clinical symptoms of allergic patients. Daily pollen count and meteorological parameters of Almería, Spain, were compiled for ten years. The clinical symptoms of 248 allergic patients were also recorded. Descriptive statistics and correlations between variables were assessed. Multivariate analyses were performed to predict the influence of meteorological factors on pollen concentration and the risk of suffer respiratory symptoms. Eight pollen families were identified as the most relevant allergenic sources. Temperature correlated with main pollen season evolution of all taxa whereas rainfall and relative humidity only correlated in Oleaceae, Pinaceae, Amaranthaceae, Asteraceae and Urticaceae. Rainfall and relative humidity were the most influential predictors of pollen concentration, except in Amaranthaceaea and Poaceae families, while temperature only influenced on Cupressaceae and Urticaceae pollen concentrations. A significant positive influence was observed between maximum temperature and rainfall with the appearance of allergic symptoms in patients sensitized to grasses, *Parietaria sp.* and *Olea sp*. This study, highlight the main aerobiological features in the region and establish a suitable tool for clinical follow-up and management of allergic patients. Further studies are needed to establish an accurate measurement aimed to control and prevent pollinosis in sensitized patients.

## 1 Introduction

Airborne pollen grains result from complex aerobiological processes that mediate the emission, dispersion, transport, and deposition of pollen. The relevance of monitoring airborne pollen concentration has been widely established, especially from a human health point of view, since between 20% and 40% of the European population suffers from pollen-induced allergies (1, 2).

Several studies have been conducted to investigate the pollen counts of different species in different geographical areas and how meteorological factors could predict pollen concentrations (3–5). However, little is known about the relationships between clinical symptoms and meteorological variables. Recently, the impact of climate change on seasonal and climatic patterns, have been suggested modifying aerobiological processes, which may influence plant spatial distributions and behaviors due to extreme weather events. These factors may change the life cycle of plants, including flowering seasonality and pollen production and be responsible for different allergenic capacities of plants by producing greater pollen concentrations, higher allergenicity, or increasing seasonality (6, 7).

The southeastern region of the Iberian Peninsula is the most semi-arid area of the European continent. Recently, progressive desertification has occurred, which favors the spread of some adapted taxa, such as *Tamarix sp.*, *Artemisia sp*., or *Urtica sp.*, that colonize new territories, becoming one of the dominant sources of seasonal allergies in mild and dry climates (8–10).

As such, we aimed to describe the aerobiological sources in the city of Almería, Spain, between 2010 and 2019, and investigate the correlation of meteorological parameters with symptoms of sensitized patients.

## 2 Material and methods

### 2.1 Area of study

The city of Almería is in the southeastern region of the Iberian Peninsula, on the Mediterranean coast. The area has a hot semi-arid climate with a high desertification impact. The average daily temperature is approximately 19.1 °C ± 5.1 °C, sunshine duration exceeds 3,000 h/year, and the annual precipitation is approximately 200 mm according to data from the State Meteorological Agency (AEMET) (http://www.aemet.es/es/portada). Almería is one of the driest locations in Europe. Local vegetation includes evergreen, deciduous, and fruit trees (e.g., olive, citrus, cypress, palms, wattles, and almond trees), and a variety of shrubs, grasses, and herbs (e.g., esparto and thyme bushes) (11).

### 2.2 Pollen collection

Daily airborne pollen levels were monitored uninterruptedly between 2010 and 2019 using a volumetric Hirst-type sampler Burkard spore-trap (Burkard Manufacturing Co., Hertfordshire, UK), which aspirates a constant flow of air (10 L/min), stationed approximately 20 m above sea level on a roof terrace (36°50´ N, 2°27´ W) in the urban center. A strip of Vaseline-coated Melinex® tape was exposed to air and changed weekly. During post-processing, the tape was cut into a 48 mm segment for each 24 h period and mounted with glycerogelatin on slides for microscopic observation. The counts were performed following the methodology approved by the Spanish Aerobiology Network (REA) (12) and the Working Group on Quality Control of the European Aerobiology Society (EAS) (13). Pollen concentrations were calculated as pollen grains/m^3^ of air (14).

The main pollen season (MPS) was established as 90% of the seasonal pollen counts, starting on the day when 5% of the total pollen was recorded and ending on the day when 95% of the total pollen was registered (15).

### 2.3 Meteorological data

Daily records of mean, minimum, and maximum temperatures (°C), rainfall (mm), relative humidity (%), and wind speed (km/h) were provided by the Almería Airport Station (AEMET), located 9 km from the pollen trap.

### 2.4 Clinical data

Clinical data were obtained from a cross-sectional observational and epidemiological study (Medical Center Dr. Zapata Yébenes; Almería, Spain) over four years (2010–2013). Skin Prick Tests (SPT) (LETI Pharma S.L.U., Madrid, Spain) were performed on patients with allergy-induced respiratory symptoms, when first referring to an allergy consultation. Allergenic symptom occurrences of 248 patients with positive SPT results for pollen, mites, profilin, and/or food allergens were recorded.

The patient population was classified according to clinical history, sensitization profile, presence of symptoms during the year, and symptom classification based on the ARIA (16) and GEMA criteria (17).

### 2.5 Statistical methods

Descriptive statistical analyses were used to detail pollen, meteorological, and clinical data. The Shapiro–Wilk test was used to determine the normal distribution of the data. The Pearson correlation coefficient was provided for normal distributions; otherwise, the Spearman correlation coefficient was calculated. Pollen season evolution was studied using correlations between meteorological parameters obtained at three different periods of time: annual data, annual data by season, and annual data by period. Generalized linear models (GLM) were used to establish the influence and impact of the temporal distribution of meteorological variables on pollen concentration by species. Finally, the prevalence of symptoms recorded in the allergic population during 2010–2013 and the local aerobiological characteristics were investigated using generalized estimating equations (GEE). *Olea sp.*, *Cupressus sp.*, grasses, *Chenopodium sp.* and/or *Salsola sp.*, *Artemisia sp.* and *Parietaria sp.* were considered. The effect size was estimated by means of odds ratios, confidence intervals, and significance levels, as detailed in the Supplementary Material.

Results with a *p*-value of < 0.05 were considered statistically significant.

All statistical analyses were performed using SAS 9.4. software (SAS Institute Inc., North Carolina, USA) and graphs were generated using GraphPad Prism 9.1.1 software (GraphPad Software, San Diego, CA, USA).

## 3 Results

### 3.1 Pollen counts and MPS

Eight pollen families were identified as the most abundant, including four arboreal taxa; Oleaceae (*Olea sp.)* (47.5%), Cupressaceae (5.1%), Pinaceae (*Pinus sp.*) (5.5%), and Fagaceae (*Quercus sp.*) (16%), and four non-arboreal taxa; Poaceae (8.8%), Amaranthaceae (9.5%), Asteraceae (*Artemisia sp.*) (2.3%), and Urticaceae (5.1%).

**Figure 1:**
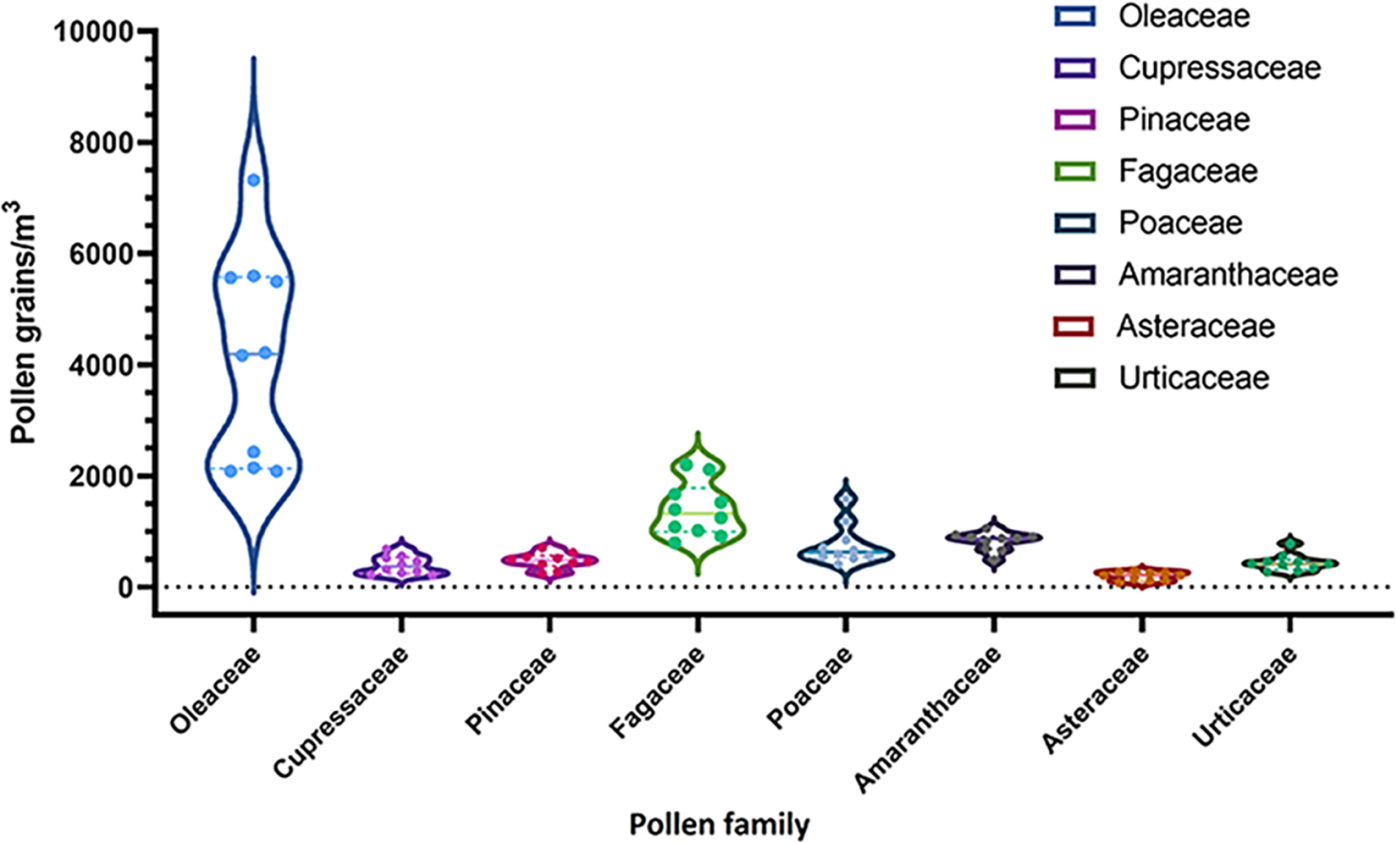
Violin graphs show total annual count of the eight main pollen families between 2010 and 2019. Interquartile ranges and median value are represented. Count of pollen as pollen grains/m^3^^.^

The total annual count of the main pollen families is shown in Figure 1, showing significant annual variability in the Oleaceae, Cupressaceae, Poaceae, and Asteraceae families.

**Figure 2:**
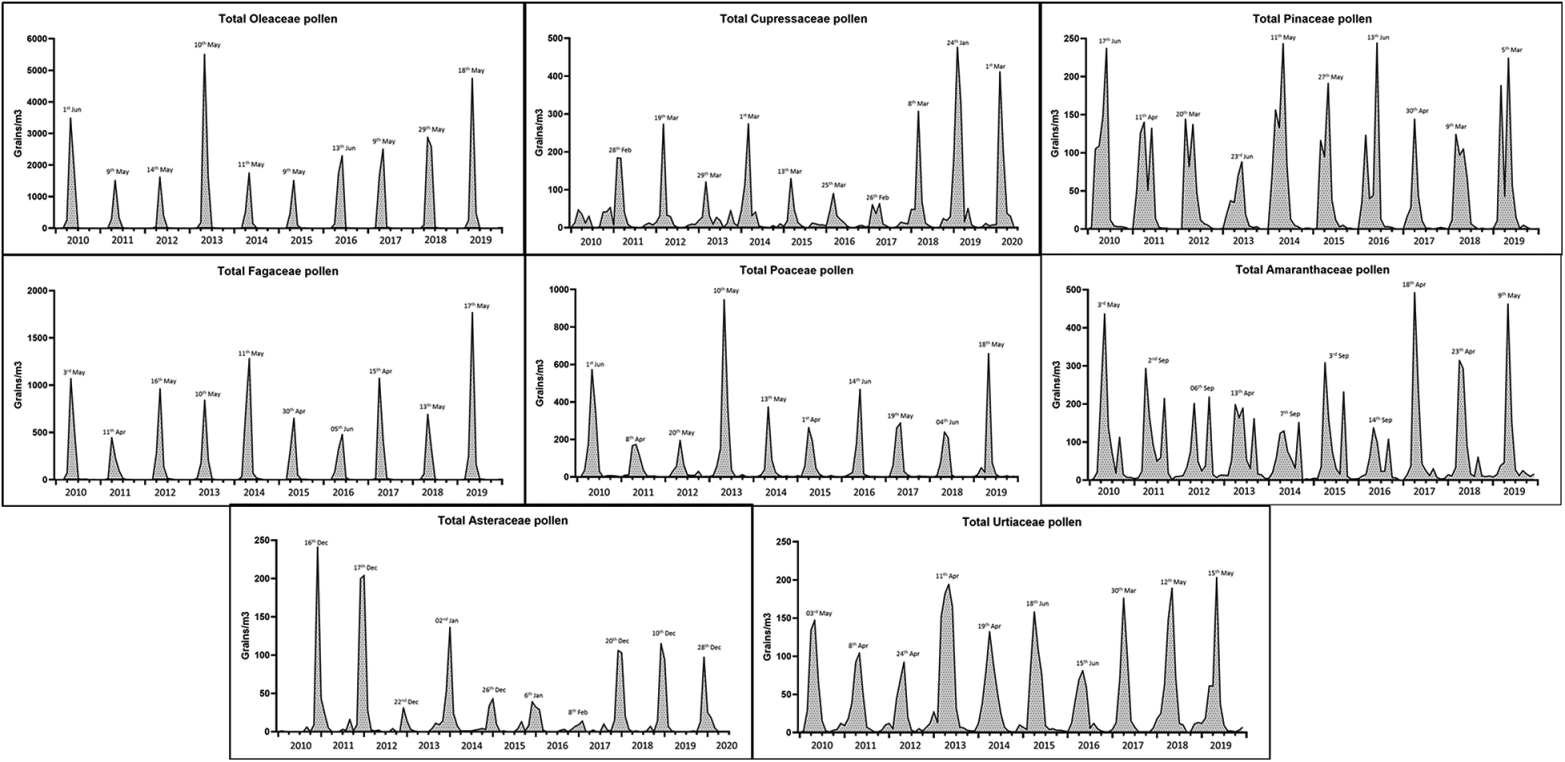
Evolution of the main pollen season of eight taxa between 2010 and 2019. Areas filled show the monthly count of pollen as pollen grains/m^3^ and peak days are represented.

Although the total annual pollen records tended to increase over the last five years of the study period (from 5,863 pollen grains/m^3^ in 2015 to 11,216 pollen grains/m^3^ in 2019), 2013 had the highest amount of total pollen (12,970 pollen grains/m^3^), especially *Olea sp.* and grass pollens, which peaked on May 10 (Figure 2). Considering the chronological appearances of airborne pollen, Cupressaceae and Asteraceae were detected during the autumn months. Oleaceae, Pinaceae, Fagaceae, and Amaranthaceae showed regular behavior in terms of the length, start, and end of their MPS during the study period. Moreover, Amaranthaceae also shows two characteristic annual peaks, that is, in spring and September, resulting in long-lasting pollen seasons. A similar duration was found in the Cupressaceae, Pinaceae, and Urticaceae families, whose MPS lasted an average of 5–6 months (Figure 2).

### 3.2 Temporal changes in meteorological data and impact on pollen concentration

**Figure 3:**
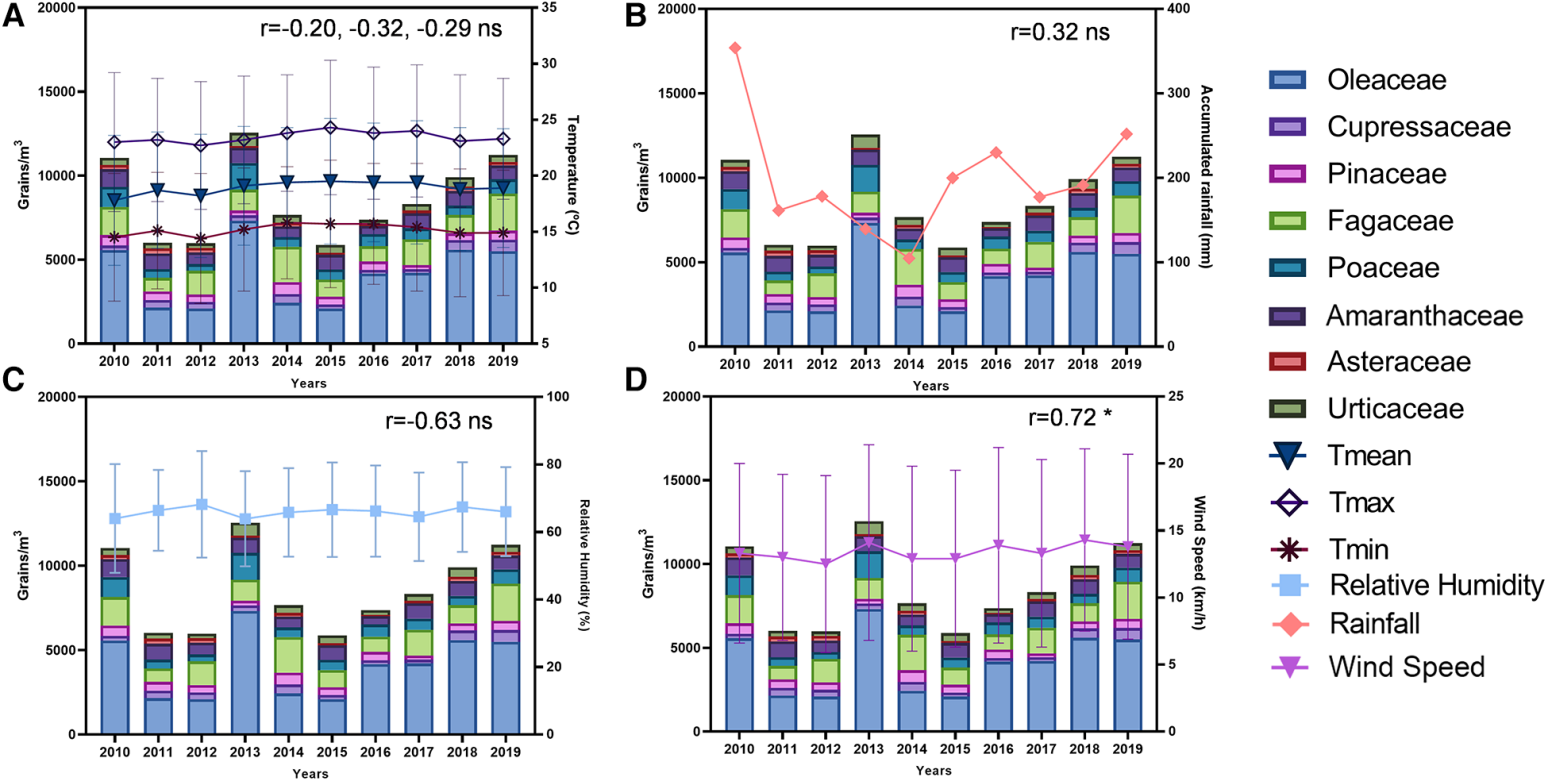
Stacked bar graphs show annual total amount of eight pollen families and meteorological parameters evolution between 2010 and 2019. (**A**) Annual mean, maximum and minimum temperatures (°C). (**B**) Annual cumulative rainfall (mm). (**C**) Annual relative humidity (%). (**D**) Annual wind speed (km/h). Count of pollen as pollen grains/m^3^. Pearsońs *r* is shown in each graph. *p < 0.05.

The annual variations of meteorological data and pollen concentration are shown in Figure 3. The annual average temperature remained constant during the study period, within 18–20 °C (Figure 3A). The cumulative annual rainfall was approximately 200 mm, with the 2010 rainfall recorded as higher than the usual, at 354 mm (Figure 3B). The average relative humidity oscillated cyclically by approximately 66% (Figure 3C), which was the same trend observed for wind speed, with a slight increase over the years (Figure 3D).

The years 2012 and 2013 had the lowest and highest amounts of pollen recorded, respectively. No significant correlations were observed between total annual pollen counts for all pollen types and annual meteorological data, except for wind speed, which was positively correlated (*r* = 0.72) (Figure 3D). When total annual pollen was considered for each individual family, significant specific correlations were observed. For example, wind speed and Oleaceae pollen levels (*r* = 0.85), relative humidity and Poaceae pollen (*r* = −0.80), minimum, mean, and maximum temperature, and Asteraceae pollen (*r* = −0.66, −0.70, and −0.66, respectively) (Supplementary Figures S1–S8).

### 3.3 Clinical data

**Figure 4:**
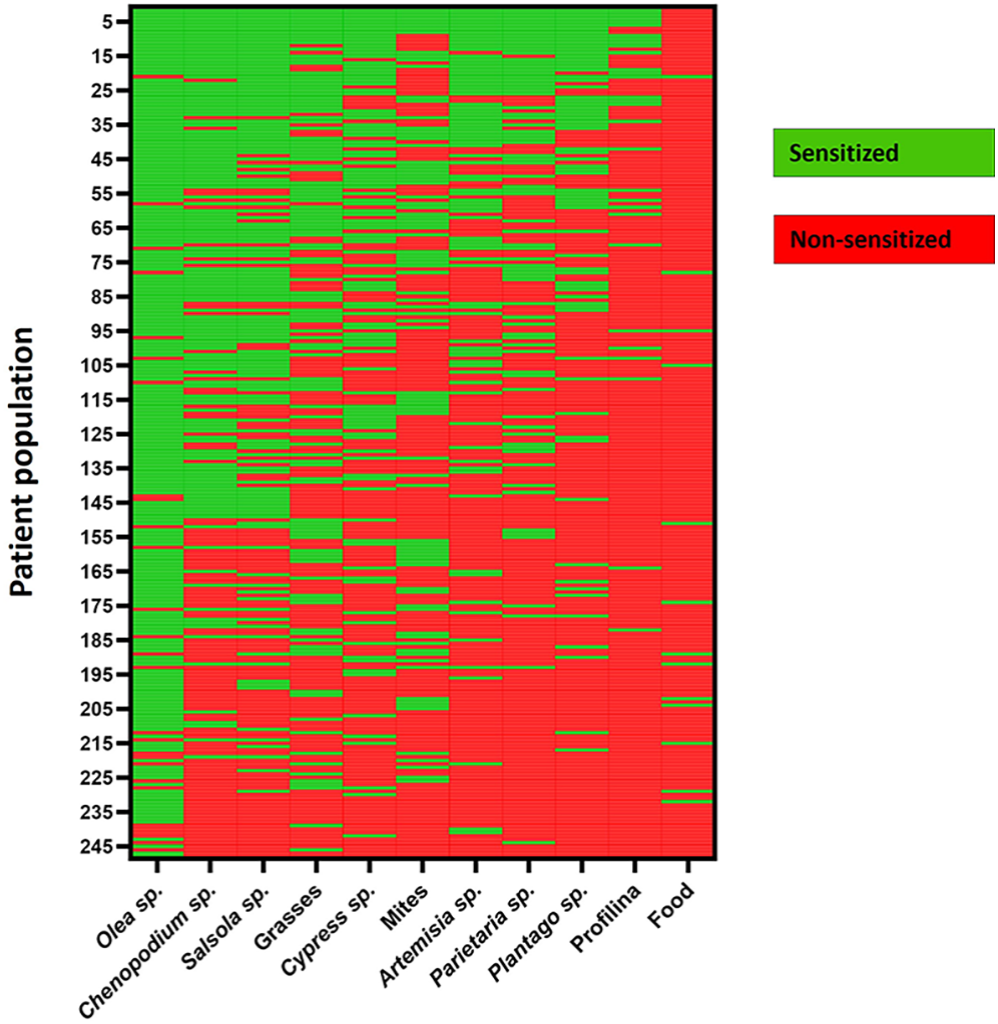
Heatmap illustrating the sensitization profiling of patients to different allergens based on skin prick test (SPT).

The clinical data are summarized in Table 1. The study population consisted of 248 patients (mean age: 32.4 ± 16.2 years; range: 1–84 years) equally distributed by sex. Almost 100% of the patients suffered from allergic respiratory symptoms, including rhinoconjunctivitis (70.2%), rhinitis (18.1%), and persistent asthma (7.6%). Patients reported symptoms during six months annually, with April, May, and June being reported by 70.6%, 92.3%, and 90.3% of affected patients, respectively. Ninety-three percent of the patients were polysensitized, with *Olea sp.* being the most prominent allergen sensitizer (88.7%), and *Chenopodium sp.* (56%), *Salsola sp.* (54.8%), grasses (47.2%), and *Cypress sp.* (41.9%) also being significant. However, profilin and food sensitization were presented in smaller percentages, that is, 12.1% and 5.2%, respectively (Figure 4).

**Table 1:** Description of clinical data categories clustered in the different years of the study: clinical history, symptom´s classification, sensitization profile and presence of symptoms during the year.

Characteristic	2010 (*N* = 74)	2011 (*N* = 58)	2012 (*N* = 59)	2013 (*N* = 57)	All (*N* = 248)
Clinical History					
Age (years)					
Mean (SD)	31.3 (15.9)	33.4 (15.7)	32.1 (16.5)	33.4 (16.8)	32.4 (16.2)
Median	32.0	36.0	32.0	32.0	32.0
Min:Max	1:81	6:76	4:71	4:84	1:84
Gender					
Male	37 (50.0%)	25 (43.1%)	28 (47.5%)	29 (50.9%)	119 (48.0%)
Female	37 (50.0%)	33 (56.9%)	31 (52.5%)	28 (49.1%)	129 (52.0%)
Family history					
Yes	30 (40.5%)	27 (46.6%)	32 (54.2%)	32 (56.1%)	121 (48.8%)
No	44 (59.5%)	31 (53.4%)	27 (45.8%)	25 (43.9%)	127 (51.2%)
Symptom´s classification*					
RC	48 (64.9%)	38 (65.5%)	40 (67.8%)	48 (84.2%)	174 (70.2%)
R	14 (18.9%)	11 (19.0%)	15 (25.4%)	5 (8.8%)	45 (18.1%)
RCA	5 (6.8%)	4 (6.9%)	2 (3.4%)	2 (3.5%)	13 (5.2%)
RCD	1 (1.4%)	3 (5.2%)	1 (1.7%)	1 (1.8%)	6 (2.4%)
RA	3 (4.1%)	1 (1.7%)	0 (0.0%)	1 (1.8%)	5 (2.0%)
RD	1 (1.4%)	1 (1.7%)	0 (0.0%)	0 (0.0%)	2 (0.8%)
C	1 (1.4%)	0 (0.0%)	0 (0.0%)	0 (0.0%)	1 (0.4%)
CA	0 (0.0%)	0 (0.0%)	1 (1.7%)	0 (0.0%)	1 (0.4%)
D	1 (1.4%)	0 (0.0%)	0 (0.0%)	0 (0.0%)	1 (0.4%)
Sensitization					
*Olea sp.*	67 (90.5%)	56 (96.6%)	49 (83.1%)	48 (84.2%)	220 (88.7%)
*Chenopodium sp.*	41 (55.4%)	38 (65.5%)	29 (49.2%)	31 (54.4%)	139 (56.0%)
*Salsola sp.*	46 (62.2%)	30 (51.7%)	32 (54.2%)	28 (49.1%)	136 (54.8%)
Grasses	34 (45.9%)	26 (44.8%)	25 (42.4%)	32 (56.1%)	117 (47.2%)
*Cypress sp.*	24 (32.4%)	32 (55.2%)	24 (40.7%)	24 (42.1%)	104 (41.9%)
Mites	24 (32.4%)	19 (32.8%)	22 (37.3%)	21 (36.8%)	86 (34.7%)
*Artemisia sp.*	24 (32.4%)	19 (32.8%)	24 (40.7%)	16 (28.1%)	83 (33.5%)
*Parietaria sp.*	23 (31.1%)	20 (34.5%)	17 (28.8%)	16 (28.1%)	76 (30.6%)
*Plantago sp.*	17 (23.0%)	25 (43.1%)	22 (37.3%)	6 (10.5%)	70 (28.2%)
Profilin	9 (12.2%)	9 (15.5%)	5 (8.5%)	7 (12.3%)	30 (12.1%)
Food	1 (1.4%)	3 (5.2%)	3 (5.1%)	6 (10.5%)	13 (5.2%)
Presence of symptoms					
January	2 (2.7%)	1 (1.7%)	4 (6.8%)	2 (3.5%)	9 (3.6%)
February	5 (6.8%)	1 (1.7%)	3 (5.1%)	1 (1.8%)	10 (4.0%)
March	11 (14.9%)	6 (10.3%)	4 (6.8%)	6 (10.5%)	27 (10.9%)
April	69 (93.2%)	46 (79.3%)	30 (50.8%)	30 (52.6%)	175 (70.6%)
May	71 (95.9%)	54 (93.1%)	51 (86.4%)	53 (93.0%)	229 (92.3%)
June	67 (90.5%)	54 (93.1%)	51 (86.4%)	52 (91.2%)	224 (90.3%)
July	5 (6.8%)	0 (0.0%)	1 (1.7%)	3 (5.3%)	9 (3.6%)
August	3 (4.1%)	0 (0.0%)	3 (5.1%)	4 (7.0%)	10 (4.0%)
September	15 (20.3%)	12 (20.7%)	17 (28.8%)	12 (21.1%)	56 (22.6%)
October	10 (13.5%)	5 (8.6%)	7 (11.9%)	3 (5.3%)	25 (10.1%)
November	5 (6.8%)	1 (1.7%)	4 (6.8%)	2 (3.5%)	12 (4.8%)
December	3 (4.1%)	0 (0.0%)	4 (6.8%)	3 (5.3%)	10 (4.0%)

* R, Rhinitis; A, Asthma; C, Conjunctivitis; D, Dermatitis.

### 3.4 Correlations between meteorological parameters and the evolution of pollen seasons

#### 3.4.1 Annual data

Statistically significant correlations between annual meteorological factors, particularly wind speed, and some parameters of pollen seasons were observed in Poaceae, Cupressaceae and Oleaceae (Table 2). Non-significant correlations were compiled in Supplementary Table S1.

**Table 2:** Correlations between annual data of meteorological factors and evolution of pollen seasons. Only significant results are shown.

Parameter	Pollen type	Meteorological factor	Coefficient	*p* value
End	Poaceae	Wind speed	−0.71 (S)	0.0217*
Duration	Poaceae	Wind speed	−0.66 (S)	0.0368*
Peak day	Cupressaceae	Wind speed	−0.87 (P)	0.0011**
	Pinaceae	Relative Humidity	−0.66 (P)	0.0378*
	Asteraceae	Mean Temperature	0.65 (S)	0.0425*
Peak concentration	Oleaceae	Wind speed	0.87 (P)	0.0010**

Type of correlation; S, Spearman; P, Pearson.

* *p* < 0.05.

** *p* < 0.01.

#### 3.4.2 Annual data by season

Generally, temperature was significantly associated with MPS characteristics, especially during the spring months in arboreal taxa. These negative correlations were also observed during the winter months with rainfall, impacting on the peaking day occurrence in Fagaceae and, duration of Pinaceae and Amaranthaceaea MPS. Positive correlations were only found in the spring months with respect to rainfall in Oleaceae, and relative humidity in Pinaceaea and Urticaceae (Table 3). Non-significant correlations were compiled in Supplementary Table S2.

**Table 3:** Correlations between annual data by season of meteorological factors and evolution of pollen seasons. Winter and spring were considered in Oleaceae, Pinaceae, Fagaceae, Poaceae, Amaranthaceae and Urticaceae. Summer and autumn were considered in Cupressaceae and Asteraceae. Only significant results are shown.

Season	Parameter	Pollen type	Meteorological factor	Coefficient	*p* value
Winter	Start	Oleaceae	Minimum temperature	−0.64 (P)	0.0457*
		Pinaceae	Mean temperature	−0.74 (S)	0.0139*
			Minimum temperature	−0.66 (S)	0.0392*
	Duration	Pinaceae	Rainfall	−0.63 (S)	0.0498*
		Fagaceae	Wind speed	−0.74 (P)	0.0145*
		Poaceae	Mean temperature	−0.67 (S)	0.0345*
		Amaranthaceae	Rainfall	−0.76 (P)	0.0108*
	Peak day	Fagaceae	Rainfall	−0.83 (S)	0.0028**
	Peak concentration	Poaceae	Mean temperature	−0.70 (S)	0.0251*
			Maximum temperature	−0.68 (S)	0.0289*
		Amaranthaceae	Minimum temperature	−0.64 (P)	0.0476*
Spring	Start	Oleaceae	Mean temperature	−0.71 (P)	0.0209*
			Maximum temperature	−0.89 (P)	0.0005***
			Minimum temperature	−0.76 (P)	0.0107*
		Fagaceae	Mean temperature	−0.72 (P)	0.0193*
	End	Oleaceae	Maximum temperature	−0.81 (S)	0.0044**
		Pinaceae	Maximum temperature	−0.70 (P)	0.0253*
			Relative humidity	0.64 (P)	0.0456*
		Fagaceae	Maximum temperature	−0.67 (P)	0.0358*
	Peak day	Oleaceae	Mean temperature	−0.73 (S)	0.0165*
			Maximum temperature	−0.63 (S)	0.0500*
			Minimum temperature	−0.71 (S)	0.0226*
		Poaceae	Minimum temperature	−0.66 (P)	0.0395*
		Amaranthaceae	Rainfall	−0.77 (S)	0.0098**
	Peak concentration	Oleaceae	Rainfall	0.67 (P)	0.0330*
		Urticaceae	Relative humidity	0.65 (P)	0.0437*
Autumn	Start	Asteraceae	Minimum temperature	−0.71 (S)	0.0205*
	Peak concentration	Asteraceae	Relative humidity	−0.70 (P)	0.0232*

Type of correlation; S, Spearman; P, Pearson.

* *p* < 0.05.

** *p* < 0.01.

*** *p* < 0.001.

#### 3.4.3 Annual data by period

When the meteorological factors were limited to short periods closer to the MPS, the statistical significance increased, confirming that temperature was the most influential parameter on the MPS evolution of each family except Fagaceae. Additionally, rainfall showed significant positive correlation with the beginning of the Asteraceae pollen season, and the end of Amaranthaceae pollen season. In the same way, relative humidity had an impact on the start and the end day in Oleaceae MPS, and duration of Urticaceae MPS (Table 4). Non-significant correlations were compiled in Supplementary Table S3.

**Table 4:** Correlations between annual data by period of meteorological factors and evolution of pollen seasons. Only significant results are shown.

Period	Parameter	Pollen type	Meteorological factor	Coefficient	*p* value
Period 1	Start	Oleaceae	Relative humidity	0.80 (P)	0.0055**
		Cupressaceae	Mean temperature	−0.93 (P)	0.0003***
			Maximum temperature	−0.92 (P)	0.0005***
			Minimum temperature	−0.92 (P)	0.0004***
		Poaceae	Mean temperature	0.70 (P)	0.0240*
			Minimum temperature	0.76 (P)	0.0102*
		Asteraceae	Mean temperature	−0.97 (S)	0.0000***
			Maximum temperature	−0.93 (S)	0.0001***
			Minimum temperature	−0.97 (S)	0.0000***
			Rainfall	0.89 (S)	0.0006***
Period 2	Peak day	Oleaceae	Maximum temperature	0.72 (S)	0.0194*
		Pinaceae	Mean temperature	0.94 (P)	0.0001***
			Maximum temperature	0.84 (P)	0.0024**
			Minimum temperature	0.93 (P)	0.0001***
		Amaranthaceae	Mean temperature	0.78 (S)	0.0080**
			Maximum temperature	0.80 (S)	0.0052**
			Minimum temperature	0.75 (S)	0.0118*
		Urticaceae	Mean temperature	0.88 (S)	0.0008***
			Maximum temperature	0.85 (P)	0.0019**
			Minimum temperature	0.73 (S)	0.0158*
	Peak concentration	Cupressaceae	Wind speed	−0.71 (P)	0.0308*
		Pinaceae	Rainfall	−0.67 (S)	0.0338*
		Poaceae	Wind speed	−0.67 (S)	0.0330*
		Asteraceae	Mean temperature	−0.67 (S)	0.0345*
			Maximum temperature	−0.69 (P)	0.0288*
Period 3	End	Oleaceae	Relative humidity	0.90 (S)	0.0004***
		Cupressaceae	Maximum temperature	0.79 (S)	0.0109*
		Amaranthaceae	Rainfall	0.63 (S)	0.0498*
Period 4	Duration	Cupressaceae	Mean temperature	0.78 (P)	0.0078**
			Maximum temperature	0.69 (P)	0.0265*
			Minimum temperature	0.80 (P)	0.0052**
		Asteraceae	Mean temperature	0.92 (P)	0.0002***
			Maximum temperature	0.93 (P)	0.0001***
			Minimum temperature	0.95 (P)	0.0000***
		Urticaceae	Relative humidity	0.63 (S)	0.0498**

Period 1; from 28 days previous to the start of pollen season to the day before the start of pollen season.

Period 2; from the day that the pollen season starts to the day before the peak day into the pollen season.

Period 3; from the peak day to the day that pollen season ends.

Period 4; the sum of period 1, 2 and 3.

Type of correlation; S, Spearman; P, Pearson.

* *p* < 0.05.

** *p* < 0.01.

*** *p* < 0.001.

### 3.5 Multivariate analysis between meteorological parameters and pollen concentration

Generalized linear model revealed significant relationships between rainfall and pollen concentrations. For each unit in which rainfall increased yearly, pollen concentrations decreased by 7.73%, 6.08%, 20.74%, 20.56%, and 6.79% compared with the reference levels of Cupressaceae, Pinaceae, Fagaceae, Asteraceae, and Urticaceae, respectively. For Oleaceae, Pinaceae, and Urticaceae, relative humidity also affected pollen concentration by a 1.44% increase, and a 0.88% and 1.73% decrease, respectively (Table 5). Additionally, the pollen concentration of Urticaceae was affected by temperature, which had the greatest influence in the case of Cupressaceae pollen, increasing almost 70% for every unit increase in annual maximum temperature (Table 5). Wind speed did not influence pollen concentration, and Amaranthaceae and Poaceae pollens were not significantly influenced by meteorological parameters.

**Table 5:** Impact of influential predictors over pollen concentration found after carrying out a negative binomial regression with stepwise selection process.

Pollen concentration	Influential Parameter	Incident Rate Ratio	IRR 95% CI lower limit	IRR 95% CI upper limit	Chi-Square	Pr > ChiSq	IRR Interpretation
Oleaceae	Relative humidity	1.0144	1.0087	1.0202	24.64	<.0001***	For every 1-unit increase in yearly Relative Humidity, pollen concentration on the reference level would increase a 1.44%
Cupressaceae	Maximum temperature	1.6852	1.4953	1.8992	73.19	<.0001***	For every 1-unit increase in yearly Maximum Temperature, pollen concentration on the reference level would increase a 68.52%
	Rainfall	0.9227	0.8908	0.9557	20.12	<.0001***	For every 1-unit increase in yearly Rainfall, pollen concentration on the reference level would decrease a 7.73%
Pinaceae	Rainfall	0.9392	0.9038	0.9759	10.29	0.0013**	For every 1-unit increase in yearly Rainfall, pollen concentration on the reference level would decrease a 6.08%
	Relative humidity	0.9912	0.9863	0.9962	11.89	0.0006***	For every 1-unit increase in yearly Relative Humidity, pollen concentration on the reference level would decrease a 0.88%
Fagaceae	Rainfall	0.7926	0.7374	0.8519	39.87	<.0001***	For every 1-unit increase in yearly Rainfall, pollen concentration on the reference level would decrease a 20.74%
Asteraceae	Rainfall	0.7944	0.6904	0.9141	10.34	0.0013**	For every 1-unit increase in yearly Rainfall, pollen concentration on the reference level would decrease a 20.56%
Urticaceae	Mean temperature	1.2273	1.0720	1.4051	8.81	0.0030**	For every 1-unit increase in yearly Mean Temperature, pollen concentration on the reference level would increase a 22.73%
	Minimum temperature	0.9067	0.8658	0.9495	17.34	<.0001***	For every 1-unit increase in yearly Minimum Temperature, pollen concentration on the reference level would decrease a 9.33%
	Rainfall	0.9321	0.8997	0.9656	15.22	<.0001***	For every 1-unit increase in yearly Rainfall, pollen concentration on the reference level would decrease a 6.79%
	Relative humidity	0.9827	0.9667	0.9990	4.33	0.0374*	For every 1-unit increase in yearly Relative Humidity, pollen concentration on the reference level would decrease a 1.73%

Only significant results are shown.

* *p* < 0.05.

** *p* < 0.01.

*** *p* < 0.001.

### 3.6 Multivariate analysis between prevalence of symptoms and aerobiological characteristics

The association between meteorological variables and pollen concentration data on patient symptomatology was also explored. The influential predictors are listed in Table 6. Maximum temperature and rainfall were responsible for increasing the odds of allergy symptoms in patients sensitized to grasses, *Parietaria sp.,* and *Olea sp.* in case of maximum temperature. Furthermore, statistical significance was observed for Amaranthaceae pollen concentrations in patients sensitized to *Chenopodium sp.* or *Salsola sp*.

**Table 6:** Impact of influential predictors over prevalence of symptoms found after carrying out a generalized estimating equations model fit with backward selection process.

Sensitization agent	Influential Parameter	Odds Ratio (OR)	OR 95% CI lower limit	OR 95% CI upper limit	Chi-Square	Pr > ChiSq	IRR Interpretation
*Olea sp.*	Maximum temperature	1.2926	1.0548	1.5840	6.12	0.0134*	An increment of 1 unit of Maximum Temperature, would increase the odds of having symptoms 1.29 times
Grasses	Mean temperature	0.5119	0.3306	0.7924	9.02	0.0027**	An increment of 1 unit of Mean Temperature, would decrease the odds of having symptoms a 48.81%
	Maximum temperature	2.4562	1.4988	4.0252	12.72	0.0004***	An increment of 1 unit of Maximum Temperature, would increase the odds of having symptoms 2.46 times
	Rainfall	1.9466	1.1977	3.1637	7.23	0.0072**	An increment of 1 unit of Rainfall, would increase the odds of having symptoms 1.95 times
*Chenopodium sp.*/ *Salsola sp.*	Amaranthaceae pollen	1.2975	1.1323	1.4867	14.05	0.0002***	An increment of 1 unit of pollen, would increase the odds of having symptoms 1.3 times
*Parietaria sp.*	Maximum temperature	2.1423	1.2441	3.6890	7.55	0.0060**	An increment of 1 unit of Maximum Temperature, would increase the odds of having symptoms 2.14 times
	Rainfall	3.5294	1.4398	8.6515	7.60	0.0058**	An increment of 1 unit of Rainfall, would increase the odds of having symptoms 3.53 times

Only significant results are shown.

* *p* < 0.05.

** *p* < 0.01.

*** *p* < 0.001.

## 4 Discussion

Clinical aerobiological studies are useful to evaluate pollen from plant species that are clinically relevant in a specific area and to improve the quality of life of pollinosis sufferers (1, 6). The semi-arid Mediterranean climate of Almería seems to be an important factor in the phenology of different taxa (8–10). Due to the known effect of pollen on human health, it is important to understand the meteorological parameters affecting the different aerobiological processes (emission, dispersion and/or transport, and deposition) of aeroallergens, and to develop the possibility of predicting them (2, 7).

Similar to other Mediterranean regions, Oleaceae is the most common airborne pollen, representing approximately half of the recorded pollen counts, followed by Fagaceae, represented by *Quercus sp* (4, 18, 19)*.* Both taxa showed short pollination periods, averaging 42 and 52 days, respectively, which coincided with the end of March to May. Other arboreal taxa, such as Cupressaceae and Pinaceae, ranked very low but extended their pollination time for a longer period. Regarding herbaceous taxa, four families dominated the pollen spectrum in the area, that is, Amaranthaceae, Poaceae, Asteraceae *(Artemisia sp.),* and Urticaceae *(Parietaria sp.)* (20), but differences between them were observed. Amaranthaceae and Urticaceae pollens showed usual annually behaviors and remained in the atmosphere for 5–6 months, consistent with reports for other areas with similar bioclimatology (8–10, 21). Poaceae and Asteraceae pollen counts showed great interannual variability, ranging between 408 and 1,587 pollen grains/m^3^ and 38–468 pollen grains/m^3^, respectively. Such variability was also observed in other families, including Oleaceae and Cupressaceae, as previously reported (8, 22). Cupressaceae and Asteraceae family pollens were the latest to appear since their pollination years do not coincide with the calendar years and were responsible for the counts recorded in autumn and the beginning of winter (9).

Regarding pollination seasonality, warmer temperatures have been shown to correlate with earlier and longer pollen seasons and higher pollen concentration (5, 6). Our results confirmed that temperature influenced the MPS characteristics related to the onset, end, and duration of arboreal taxa. Mild winter temperatures correlated with earlier start days in the cases of *Olea sp.* and Pinaceae. Similarly, when temperatures remained mild during the spring months, the Oleaceae, Pinaceae, and Fagaceae MPSs began and ended earlier. These trends have been previously observed in other Mediterranean regions (18, 19). Regarding pollen concentrations, we found that records in grasses and Amaranthaceae species were lower when temperatures increased during winter. Additionally, the closer to the MPS, the stronger correlation with temperature, which also affects the herbaceous taxa. The Cupressaceae and Asteraceae pollen seasons began earlier and lasted longer when temperatures from the previous days to the start day were mild and continued to be so. However, the opposite effect was observed in Poaceae, causing a delay to the start of the pollen season. Once the Oleaceae, Pinaceae, Amaranthaceae, and Urticaceae seasons started, the peak day was delayed, and the peak concentration was lower for Asteraceae when temperatures increased. According to the results of our multivariate analyses, temperature was not found to be an influential predictor of pollen concentration, contrary to other similar models (23), we only found this possible prediction with Cupressaceae and Urticaceae pollen concentration.

Precipitation had varying effects on pollen season timing. Negative correlations between rainfall and MPS duration in Pinaceae and Amaranthaceae and the peak day in Fagaceae were recorded in winter. This negative correlation was also observed between rainfall and Amaranthaceae peak day in spring. However, positive correlations between rainfall prior to the beginning or end of the MPS and the start and end days were observed in Asteraceae and Amaranthaceae, respectively. In our study area, have been described an increased presence of Amaranthaceae, being strongly influenced by rainfall (10, 21). Increased precipitation may have a short-term effect causing low pollen concentrations potentially due to the “wash out” effect (6). Our multivariate analyses suggested this effect was present in five of the eight main pollen families in the region, with rainfall being an influential predictor of Cupressaceae, Pinaceae, Fagaceae, Asteraceae, and Urticaceae pollen concentrations. Notably, no statistical significance was found between rainfall and the evolution of grass pollen seasons or records.

Regarding the relative humidity and evolution of pollen seasons, a few significant correlations were observed. Notably, the relative humidity prior to the start and end of MPS revealed strong positive correlations with these parameters in Oleaceae. Similarly, the Urticaceae MPS lasted longer when the relative humidity was high. Previous researchers have suggested that days with rain or high relative humidity make pollen grains heavier and settle, favoring lower pollen concentrations (24). Further, we found that relative humidity decreased Pinaceae and Urticaceae pollen concentrations, as seen before rainfall, but increased Oleaceae. This effect was also previously observed in some herbaceous taxa, such us Urticaceae and Amaranthaceae (23).

The wind speed was the least influential meteorological factor; however, it had a considerable influence on the end date and duration of the pollen seasons of grasses, over the peak day of Cupressaceae, and peak concentration of Oleaceae. Although wind speed was the only meteorological parameter with a significant correlation with total pollen recorded in 2013, the multivariate model did not reveal it as an influential predictor of pollen concentration, potentially due to the orientation and altitude of the pollen trap.

Knowledge of pollen counts, and the influence of meteorological parameters can be used in the management of allergy patients in this region. Warmer temperatures are associated with earlier, longer, and more intense pollen seasons, which have potential implications for allergic populations (6, 25). Similarly, higher aeroallergen concentrations are associated with increased humidity and precipitation due to pollen grain rupturing, which releases thousands of allergenic particles into the air and elicits acute reactions in sensitized individuals (18, 26). Almost all the patients in our study were sensitized to olive pollen, and all reported symptoms during April, May, and June, coinciding with the Oleaceae, but also Poaceae and Urticaceae MPS during a period of considerable allergological interest (2). Patients sensitized to these pollen types would increase the odds of having symptoms when the maximum temperature and rainfall (not in Oleaceae) increased by 1 unit. In fact, these weather conditions have been linked to a high risk of allergic symptoms for these patients in the spring and summer months (25, 27). However, it could be relevant for future studies, to select participants with no other relevant sensitization to reveal specific correlations in order to draw clear conclusions (28). In half of the sensitized patients, Amaranthaceae, including *Chenopodium sp.* and *Salsola sp.,* was the second family with clinical importance in Almería, and special attention should be paid to this pollen type in September and October, coinciding with the second pollination peak (10). Nevertheless, no meteorological parameters were found to influence the symptom risk. Even though Cupressaceae and Asteraceae pollen, represented by *Artemisia sp*., contributed the least to the total annual counts, they must be considered because there was a significant prevalence in the allergic population (42% and 34%, respectively). Nonetheless, none of the meteorological factors influenced symptoms prevalence, likely because the levels detected in both taxa did not reach the activation threshold, as we could see in the low percentages of symptoms reported during autumn and winter. Moreover, there was not sufficient Cupressaceae representation in the study area, leading to the conclusion that cypress pollen in Almería could be considered proximity pollen. One limitation of our study was the lack of information regarding the population sensitized to Fagaceae, represented by *Quercus sp*, and Pinaceae pollen families, and the rest of the years of the aerobiological study (2015–2019) regarding other allergenic sources.

## 5 Conclusions

The main pollen contributing taxa were Oleaceae, Fagaceae, Amaranthaceae, Poaceae, Pinaceae, Cupressaceae, Urticaceae and Asteraceae. Our study shows that temperature influences the main pollen season onset, end, and duration characteristics, whereas relative humidity and rainfall primarily affect airborne pollen concentrations in arboreal taxa. There are no clear trends in herbaceous taxa because of individual variability. The main sources of sensitization in the study area are *Olea sp.*, *Chenopodium sp.*, *Salsola sp.*, grasses, and *Cypress sp.*, with great importance of polysensitized patients and presence of allergic symptoms during the spring months. Maximum temperature and rainfall are the most influential predictors of symptoms prevalence, particularly in patients sensitized to *Olea sp.,* grasses, and *Parietaria sp*. This study highlights the main aerobiological features of European semi-desert area and establishes a suitable tool for the clinical follow-up and management of allergic patients and opens the possibility of establishing predictive factors for use in public health and preventive allergology.

## Data Availability

The original contributions presented in the study are included in the article/**Supplementary Material**, further inquiries can be directed to the corresponding author.
