# Supplementary material for: Aerobiological and clinical study in the semidesertic area of the Southeastern of Spain
Source: Front Allergy. 2024 Mar 25;5:1328940. doi: 10.3389/falgy.2024.1328940 (PMC10999673; doi:10.3389/falgy.2024.1328940)
Supplement: Supplementary file 1 [file Datasheet1.pdf]

Supplementary material.

Material and methods:

Statistical methods

Generalized linear models (GLM) were performed as follows. Briefly, the distribution of the response variable (pollen concentration) was checked against the pollen concentration count, as a count variable of the Poisson distribution family. Annual and monthly trends were explored using generalized additive models (GAMs) to evaluate the yearly and monthly linear and non-linear trends of pollen concentrations (data not shown). Preselection of the most influential meteorological and temporal predictors over pollen concentrations fit a GLM with all possible predictors as input and applying a stepwise model selection method. Preselected predictors were included in the final model to assess their quantitative effects on pollen concentration by GLM.

Generalized estimating equations (GEE) were calculated as follows. Briefly, for all sensitization agents, the presence of symptoms was considered a binary outcome. Logistic regression was used to model binomial response variables, considering repeated measurements for each patient (presence/absence of symptoms every month). To explore the linear trend of the response variable, the GEE model was fitted for each sensitizing agent with year and month as explanatory variables, and the presence of symptoms as a response variable distributed as binomial. Then, the most influential predictors over the presence of symptoms were preselected by applying the backward method, and finally, the preselected predictors were included in a model to assess their quantitative effects on the response variable by means of GEE.

Table S1.A. Correlations between annual data of meteorological factors and the start of pollen seasons. Only non-significant results are shown.

Pollen type	Meteorological factor	Coefficient	Pollen type	Meteorological factor	Coefficient
Oleaceae	Mean Temperature	-0.38 (P)	Poaceae	Mean Temperature	0.23 (P)
	Maximum Temperature	-0.56 (P)		Maximum Temperature	0.18 (P)
	Minimum Temperature	-0.51 (P)		Minimum Temperature	0.42 (P)
	Rainfall	0.27 (P)		Rainfall	-0.15 (P)
	Relative Humidity	0.30 (P)		Relative Humidity	0.04 (P)
	Wind speed	0.49 (P)		Wind speed	0.24 (P)
Cupressaceae	Mean Temperature	0.09 (P)	Amaranthaceae	Mean Temperature	-0.43 (P)
	Maximum Temperature	-0.02 (P)		Maximum Temperature	-0.11 (P)
	Minimum Temperature	-0.07 (P)		Minimum Temperature	-0.36 (P)
	Rainfall	0.27 (P)		Rainfall	0.59 (P)
	Relative Humidity	0.01 (P)		Relative Humidity	-0.29 (P)
	Wind speed	0.37 (P)		Wind speed	-0.10 (P)
Pinaceae	Mean Temperature	-0.40 (S)	Asteraceae	Mean Temperature	-0.21 (S)
	Maximum Temperature	-0.31 (S)		Maximum Temperature	-0.03 (S)
	Minimum Temperature	-0.53 (S)		Minimum Temperature	-0.26 (S)
	Rainfall	0.47 (S)		Rainfall	0.54 (S)
	Relative Humidity	0.61 (S)		Relative Humidity	-0.13 (S)
	Wind speed	-0.50 (S)		Wind speed	0.06 (S)
Fagaceae	Mean Temperature	-0.42 (P)	Urticaceae	Mean Temperature	0.19 (S)
	Maximum Temperature	-0.37 (P)		Maximum Temperature	0.15 (S)
	Minimum Temperature	-0.28 (P)		Minimum Temperature	0.03 (S)
	Rainfall	0.39 (P)		Rainfall	0.50 (S)
	Relative Humidity	0.26 (P)		Relative Humidity	-0.19 (S)
	Wind speed	0.19 (P)		Wind speed	0.12 (S)

Type of correlation; S: Spearman, P: Pearson.

Table S1.B. Correlations between annual data of meteorological factors and the end of pollen seasons. Only non-significant results are shown.

Pollen type	Meteorological factor	Coefficient	Pollen type	Meteorological factor	Coefficient
Oleaceae	Mean Temperature	-0.37 (S)	Poaceae	Mean Temperature	-0.32 (S)
	Maximum Temperature	-0.47 (S)		Maximum Temperature	-0.30 (S)
	Minimum Temperature	-0.27 (S)		Minimum Temperature	-0.04 (S)
	Rainfall	-0.10 (S)		Rainfall	-0.48 (S)
	Relative Humidity	0.06 (S)		Relative Humidity	0.33 (S)
	Wind speed	0.56 (S)			
Cupressaceae	Mean Temperature	0.55 (P)	Amaranthaceae	Mean Temperature	-0.37 (P)
	Maximum Temperature	0.45 (P)		Maximum Temperature	-0.50 (P)
	Minimum Temperature	0.56 (P)		Minimum Temperature	-0.40 (P)
	Rainfall	-0.46 (P)		Rainfall	0.14 (P)
	Relative Humidity	0.30 (P)		Relative Humidity	0.23 (P)
	Wind speed	0.01 (P)		Wind speed	-0.12 (P)
Pinaceae	Mean Temperature	-0.31 (P)	Asteraceae	Mean Temperature	0.42 (P)
	Maximum Temperature	-0.46 (P)		Maximum Temperature	0.54 (P)
	Minimum Temperature	-0.28 (P)		Minimum Temperature	0.52 (P)
	Rainfall	-0.03 (P)		Rainfall	0.09 (P)
	Relative Humidity	0.06 (P)		Relative Humidity	-0.48 (P)
	Wind speed	0.02 (P)		Wind speed	0.05 (P)
Fagaceae	Mean Temperature	-0.51 (P)	Urticaceae	Mean Temperature	-0.60 (S)
	Maximum Temperature	-0.57 (P)		Maximum Temperature	-0.60 (S)
	Minimum Temperature	-0.42 (P)		Minimum Temperature	-0.49 (S)
	Rainfall	0.34 (P)		Rainfall	0.05 (S)
	Relative Humidity	0.25 (P)		Relative Humidity	0.56 (S)
	Wind speed	0.27 (P)		Wind speed	0.03 (S)

Type of correlation; S: Spearman, P: Pearson.

Table S1.C. Correlations between annual data of meteorological factors and the duration of pollen seasons. Only non-significant results are shown.

Pollen type	Meteorological factor	Coefficient	Pollen type	Meteorological factor	Coefficient
Oleaceae	Mean Temperature	-0.28 (S)	Poaceae	Mean Temperature	-0.40 (S)
	Maximum Temperature	-0.16 (S)		Maximum Temperature	-0.29 (S)
	Minimum Temperature	0.18 (S)		Minimum Temperature	-0.39 (S)
	Rainfall	-0.38 (S)		Rainfall	-0.07 (S)
	Relative Humidity	-0.20 (S)		Relative Humidity	0.30 (S)
	Wind speed	-0.13 (S)			
Cupressaceae	Mean Temperature	0.16 (P)	Amaranthaceae	Mean Temperature	0.03 (P)
	Maximum Temperature	0.20 (P)		Maximum Temperature	-0.29 (P)
	Minimum Temperature	0.29 (P)		Minimum Temperature	-0.04 (P)
	Rainfall	-0.40 (P)		Rainfall	-0.31 (P)
	Relative Humidity	0.12 (P)		Relative Humidity	0.37 (P)
	Wind speed	-0.28 (P)		Wind speed	-0.02 (P)
Pinaceae	Mean Temperature	0.11 (P)	Asteraceae	Mean Temperature	0.36 (P)
	Maximum Temperature	-0.22 (P)		Maximum Temperature	0.21 (P)
	Minimum Temperature	0.09 (P)		Minimum Temperature	0.39 (P)
	Rainfall	-0.37 (P)		Rainfall	-0.27 (P)
	Relative Humidity	-0.22 (P)		Relative Humidity	0.20 (P)
	Wind speed	0.38 (P)		Wind speed	-0.07 (P)
Fagaceae	Mean Temperature	-0.17 (P)	Urticaceae	Mean Temperature	-0.60 (S)
	Maximum Temperature	-0.32 (P)		Maximum Temperature	-0.58 (S)
	Minimum Temperature	-0.22 (P)		Minimum Temperature	-0.42 (S)
	Rainfall	-0.03 (P)		Rainfall	-0.35 (S)
	Relative Humidity	0.02 (P)		Relative Humidity	0.56 (S)
	Wind speed	0.13 (P)		Wind speed	-0.10 (S)

Type of correlation; S: Spearman, P: Pearson.

Table S1.D. Correlations between annual data of meteorological factors and the peak day of pollen seasons. Only non-significant results are shown.

Pollen type	Meteorological factor	Coefficient	Pollen type	Meteorological factor	Coefficient
Oleaceae	Mean Temperature	-0.39 (S)	Poaceae	Mean Temperature	-0.24 (P)
	Maximum Temperature	-0.41 (S)		Maximum Temperature	-0.31 (P)
	Minimum Temperature	-0.37 (S)		Minimum Temperature	-0.29 (P)
	Rainfall	0.56 (S)		Rainfall	0.34 (P)
	Relative Humidity	0.03 (S)		Relative Humidity	-0.09 (P)
	Wind speed	0.48 (S)		Wind speed	0.49 (P)
Cupressaceae	Mean Temperature	0.04 (P)	Amaranthaceae	Mean Temperature	0.09 (S)
	Maximum Temperature	0.18 (P)		Maximum Temperature	0.18 (S)
	Minimum Temperature	0.16 (P)		Minimum Temperature	0.26 (S)
	Rainfall	-0.22 (P)		Rainfall	0.02 (S)
	Relative Humidity	0.12 (P)		Relative Humidity	0.50 (S)
				Wind speed	-0.52 (S)
Pinaceae	Mean Temperature	0.16 (P)	Asteraceae		
	Maximum Temperature	0.35 (P)		Maximum Temperature	0.56 (S)
	Minimum Temperature	0.38 (P)		Minimum Temperature	0.60 (S)
	Rainfall	0.12 (P)		Rainfall	0.01 (S)
				Relative Humidity	-0.09 (S)
	Wind speed	0.05 (P)		Wind speed	-0.08 (S)
Fagaceae	Mean Temperature	0.04 (P)	Urticaceae	Mean Temperature	0.23 (P)
	Maximum Temperature	-0.15 (P)		Maximum Temperature	0.32 (P)
	Minimum Temperature	0.00 (P)		Minimum Temperature	0.24 (P)
	Rainfall	0.13 (P)		Rainfall	0.37 (P)
	Relative Humidity	0.27 (P)		Relative Humidity	0.35 (P)
	Wind speed	0.39 (P)		Wind speed	0.17 (P)

Type of correlation; S: Spearman, P: Pearson.

Table S1.E. Correlations between annual data of meteorological factors and the peak concentration of pollen seasons. Only non-significant results are shown.

Pollen type	Meteorological factor	Coefficient	Pollen type	Meteorological factor	Coefficient
Oleaceae	Mean Temperature	0.06 (P)	Poaceae	Mean Temperature	0.35 (S)
	Maximum Temperature	-0.14 (P)		Maximum Temperature	0.46 (S)
	Minimum Temperature	-0.08 (P)		Minimum Temperature	0.31 (S)
	Rainfall	0.12 (P)		Rainfall	0.23 (S)
	Relative Humidity	-0.34 (P)		Relative Humidity	-0.40 (S)
				Wind speed	0.33 (S)
Cupressaceae	Mean Temperature	-0.46 (P)	Amaranthaceae	Mean Temperature	0.35 (P)
	Maximum Temperature	-0.37 (P)		Maximum Temperature	0.46 (P)
	Minimum Temperature	-0.56 (P)		Minimum Temperature	0.31 (P)
	Rainfall	0.51 (P)		Rainfall	0.23 (P)
	Relative Humidity	-0.30 (P)		Relative Humidity	-0.40 (P)
	Wind speed	0.21 (S)		Wind speed	0.33 (P)
Pinaceae	Mean Temperature	-0.08 (S)	Asteraceae	Mean Temperature	-0.49 (P)
	Maximum Temperature	0.12 (S)		Maximum Temperature	-0.36 (P)
	Minimum Temperature	0.16 (S)		Minimum Temperature	-0.48 (P)
	Rainfall	0.09 (S)		Rainfall	0.43 (P)
	Relative Humidity	0.30 (S)		Relative Humidity	-0.48 (P)
	Wind speed	-0.21 (S)		Wind speed	0.11 (P)
Fagaceae	Mean Temperature	-0.07 (P)	Urticaceae	Mean Temperature	0.36 (P)
	Maximum Temperature	-0.30 (P)		Maximum Temperature	0.16 (P)
	Minimum Temperature	-0.10 (P)		Minimum Temperature	0.14 (P)
	Rainfall	-0.58 (P)		Rainfall	-0.21 (P)
	Relative Humidity	-0.05 (P)		Relative Humidity	-0.35 (P)
	Wind speed	-0.16 (P)		Wind speed	0.55 (P)

Type of correlation; S: Spearman, P: Pearson.

Table S2.A1. Correlations between annual data by season of meteorological factors and the start of pollen seasons. Winter and spring were considered in Oleaceae, Pinaceae, Fagaceae, Poaceae, Amaranthaceae and Urticaceae. Only non-significant results are shown.

	Winter (Dec-Jan-Feb)			Spring (Mar-Apr-May)	
**Pollen type**	**Meteorological factor**	**Coefficient**	**Pollen type**	**Meteorological factor**	**Coefficient**
Oleaceae	Mean Temperature	-0.29 (P)	Oleaceae		
	Maximum Temperature	-0.15 (P)			
	Rainfall	-0.46 (P)		Rainfall	0.16 (P)
	Relative Humidity	-0.16 (P)		Relative Humidity	0.58 (P)
	Wind speed	-0.18 (S)		Wind speed	0.37 (P)
Pinaceae			Pinaceae	Mean Temperature	-0.42 (S)
	Maximum Temperature	-0.48 (S)		Maximum Temperature	0.08 (S)
				Minimum Temperature	-0.41 (S)
	Rainfall	0.19 (S)		Rainfall	-0.12 (S)
	Relative Humidity	-0.40 (S)		Relative Humidity	0.14 (S)
	Wind speed	-0.17 (S)		Wind speed	-0.18 (S)
Fagaceae	Mean Temperature	-0.38 (P)	Fagaceae		
	Maximum Temperature	-0.23 (P)		Maximum Temperature	-0.62 (P)
	Minimum Temperature	-0.40 (P)		Minimum Temperature	-0.60 (P)
	Rainfall	-0.17 (P)		Rainfall	-0.07 (P)
	Relative Humidity	0.22 (P)		Relative Humidity	0.39 (P)
	Wind speed	0.23 (P)		Wind speed	0.10 (P)
Poaceae	Mean Temperature	0.32 (P)	Poaceae	Mean Temperature	0.00 (P)
	Maximum Temperature	0.32 (P)		Maximum Temperature	-0.13 (P)
	Minimum Temperature	0.41 (P)		Minimum Temperature	0.05 (P)
	Rainfall	-0.19 (P)		Rainfall	-0.30 (P)
	Relative Humidity	0.46 (P)		Relative Humidity	-0.07 (P)
	Wind speed	0.36 (P)		Wind speed	0.12 (P)
Amaranthaceae	Mean Temperature	-0.41 (P)	Amaranthaceae	Mean Temperature	-0.37 (P)
	Maximum Temperature	-0.40 (P)		Maximum Temperature	-0.14 (P)
	Minimum Temperature	-0.16 (P)		Minimum Temperature	-0.25 (P)
	Rainfall	0.61 (P)		Rainfall	0.43 (P)
	Relative Humidity	0.41 (P)		Relative Humidity	0.23 (P)
	Wind speed	-0.14 (P)		Wind speed	-0.16 (P)
Urticaceae	Mean Temperature	-0.05 (S)	Urticaceae	Mean Temperature	-0.44 (S)
	Maximum Temperature	-0.23 (S)		Maximum Temperature	-0.32 (S)
	Minimum Temperature	0.19 (S)		Minimum Temperature	-0.23 (S)
	Rainfall	0.32 (S)		Rainfall	0.21 (S)
	Relative Humidity	0.27 (S)		Relative Humidity	0.53 (S)
	Wind speed	0.41 (S)		Wind speed	-0.33 (S)

Type of correlation; S: Spearman, P: Pearson.

Table S2.A2. Correlations between annual data by season of meteorological factors and the start of pollen seasons. Summer and autumn were considered in Cupressaceae and Asteraceae. Only non-significant results are shown.

Summer (Jun-Jul-Aug)			Autumn (Sep-Oct-Nov)		
**Pollen type**	**Meteorological factor**	**Coefficient**	**Pollen type**	**Meteorological factor**	**Coefficient**
Cupressaceae	Mean Temperature	-0.19 (P)	Cupressaceae	Mean Temperature	0.06 (P)
	Maximum Temperature	-0.30 (P)		Maximum Temperature	-0.00 (P)
	Minimum Temperature	-0.31 (P)		Minimum Temperature	-0.00 (P)
	Rainfall	-0.00 (P)		Rainfall	0.38 (P)
	Relative Humidity	0.25 (P)		Relative Humidity	-0.25 (P)
	Wind speed	0.34 (S)		Wind speed	0.31 (P)
Asteraceae	Mean Temperature	0.12 (S)	Asteraceae	Mean Temperature	-0.61 (S)
	Maximum Temperature	0.15 (S)		Maximum Temperature	-0.35 (S)
	Minimum Temperature	-0.03 (S)			
	Rainfall	0.02 (S)		Rainfall	0.45 (S)
	Relative Humidity	-0.24 (S)		Relative Humidity	-0.27 (S)
	Wind speed	0.15 (S)		Wind speed	0.27 (S)

Type of correlation; S: Spearman, P: Pearson.

Table S2.B1. Correlations between annual data by season of meteorological factors and the end of pollen seasons. Winter and spring were considered in Oleaceae, Pinaceae, Fagaceae, Poaceae, Amaranthaceae and Urticaceae. Only non-significant results are shown.

Winter (Dec-Jan-Feb)			Spring (Mar-Apr-May)		
**Pollen type**	**Meteorological factor**	**Coefficient**	**Pollen type**	**Meteorological factor**	**Coefficient**
Oleaceae	Mean Temperature	-0.05 (S)	Oleaceae	Mean Temperature	-0.52 (S)
	Maximum Temperature	-0.14 (S)			
	Minimum Temperature	-0.27 (S)		Minimum Temperature	-0.41 (S)
	Rainfall	-0.54 (S)		Rainfall	0.29 (S)
	Relative Humidity	0.24 (S)		Relative Humidity	0.41 (S)
	Wind speed	-0.51 (S)		Wind speed	0.44 (S)
Pinaceae	Mean Temperature	-0.21 (P)	Pinaceae	Mean Temperature	-0.51 (P)
	Maximum Temperature	-0.13 (P)			
	Minimum Temperature	-0.43 (P)		Minimum Temperature	-0.37 (P)
	Rainfall	-0.42 (S)		Rainfall	-0.14 (P)
	Relative Humidity	-0.20 (P)			
	Wind speed	-0.29 (P)		Wind speed	-0.07 (P)
Fagaceae	Mean Temperature	-0.41 (P)	Fagaceae	Mean Temperature	-0.62 (P)
	Maximum Temperature	-0.24 (P)			
	Minimum Temperature	-0.47 (P)		Minimum Temperature	-0.52 (P)
	Rainfall	-0.20 (S)		Rainfall	0.12 (P)
	Relative Humidity	0.35 (P)		Relative Humidity	0.31 (P)
	Wind speed	-0.32 (P)		Wind speed	0.28 (P)
Poaceae	Mean Temperature	-0.48 (S)	Poaceae	Mean Temperature	-0.03 (S)
	Maximum Temperature	-0.45 (S)		Maximum Temperature	0.15 (S)
	Minimum Temperature	-0.25 (S)		Minimum Temperature	0.10 (S)
	Rainfall	0.25 (S)		Rainfall	-0.41 (S)
	Relative Humidity	-0.09 (S)		Relative Humidity	-0.08 (S)
	Wind speed	-0.09 (S)		Wind speed	-0.07 (S)
Amaranthaceae	Mean Temperature	-0.31 (P)	Amaranthaceae	Mean Temperature	-0.42 (P)
	Maximum Temperature	-0.08 (P)		Maximum Temperature	-0.47 (P)
	Minimum Temperature	-0.54 (P)		Minimum Temperature	-0.32 (P)
	Rainfall	-0.41 (S)		Rainfall	-0.19 (P)
	Relative Humidity	-0.30 (P)		Relative Humidity	0.34 (P)
	Wind speed	-0.50 (P)		Wind speed	-0.19 (P)
Urticaceae	Mean Temperature	-0.36 (S)	Urticaceae	Mean Temperature	-0.60 (S)
	Maximum Temperature	-0.30 (S)		Maximum Temperature	-0.42 (S)
	Minimum Temperature	-0.54 (S)		Minimum Temperature	-0.62 (S)
	Rainfall	-0.33 (S)		Rainfall	-0.14 (S)
	Relative Humidity	0.21 (S)		Relative Humidity	-0.05 (S)
	Wind speed	-0.45 (S)		Wind speed	0.19 (S)

Type of correlation; S: Spearman, P: Pearson.

Table S2.B2. Correlations between annual data by season of meteorological factors and the end of pollen seasons. Summer and autumn were considered in Cupressaceae and Asteraceae. Only non-significant results are shown.

Summer (Jun-Jul-Aug)			Autumn (Sep-Oct-Nov)		
**Pollen type**	**Meteorological factor**	**Coefficient**	**Pollen type**	**Meteorological factor**	**Coefficient**
Cupressaceae	Mean Temperature	0.32 (P)	Cupressaceae	Mean Temperature	0.45 (S)
	Maximum Temperature	0.31 (P)		Maximum Temperature	0.45 (P)
	Minimum Temperature	0.24 (P)		Minimum Temperature	0.44 (P)
	Rainfall	0.09 (S)		Rainfall	-0.17 (P)
	Relative Humidity	0.12 (P)		Relative Humidity	0.18 (P)
	Wind speed	0.10 (P)		Wind speed	0.48 (P)
Asteraceae	Mean Temperature	0.60 (P)	Asteraceae	Mean Temperature	-0.05 (S)
	Maximum Temperature	0.59 (P)		Maximum Temperature	0.26 (P)
	Minimum Temperature	0.56 (P)		Minimum Temperature	-0.05 (P)
	Rainfall	0.06 (S)		Rainfall	-0.20 (P)
	Relative Humidity	-0.62 (P)		Relative Humidity	-0.14 (P)
	Wind speed	0.34 (P)		Wind speed	-0.21 (P)

Type of correlation; S: Spearman, P: Pearson.

Table S2.C1. Correlations between annual data by season of meteorological factors and the duration of pollen seasons. Winter and spring were considered in Oleaceae, Pinaceae, Fagaceae, Poaceae, Amaranthaceae and Urticaceae. Only non-significant results are shown.

Winter (Dec-Jan-Feb)			Spring (Mar-Apr-May)		
**Pollen type**	**Meteorological factor**	**Coefficient**	**Pollen type**	**Meteorological factor**	**Coefficient**
Oleaceae	Mean Temperature	-0.02 (S)	Oleaceae	Mean Temperature	0.04 (S)
	Maximum Temperature	-0.01 (S)		Maximum Temperature	-0.02 (S)
	Minimum Temperature	0.14 (S)		Minimum Temperature	0.18 (S)
	Rainfall	0.06 (S)		Rainfall	-0.15 (S)
	Relative Humidity	0.59 (S)		Relative Humidity	-0.19 (S)
	Wind speed	0.01 (S)		Wind speed	-0.09 (S)
Pinaceae	Mean Temperature	0.32 (P)	Pinaceae	Mean Temperature	-0.05 (P)
	Maximum Temperature	0.27 (P)		Maximum Temperature	-0.57 (S)
	Minimum Temperature	0.06 (P)		Minimum Temperature	-0.01 (P)
				Rainfall	-0.04 (P)
	Relative Humidity	-0.01 (P)		Relative Humidity	0.45 (P)
	Wind speed	-0.24 (P)		Wind speed	0.22 (P)
Fagaceae	Mean Temperature	-0.09 (P)	Fagaceae	Mean Temperature	0.06 (P)
	Maximum Temperature	-0.03 (P)		Maximum Temperature	-0.12 (P)
	Minimum Temperature	-0.13 (P)		Minimum Temperature	0.04 (P)
	Rainfall	-0.43 (S)		Rainfall	0.26 (P)
	Relative Humidity	0.21 (P)		Relative Humidity	-0.08 (P)
				Wind speed	0.27 (P)
Poaceae			Poaceae	Mean Temperature	-0.05 (S)
	Maximum Temperature	-0.44 (S)		Maximum Temperature	0.26 (S)
	Minimum Temperature	-0.48 (S)		Minimum Temperature	-0.02 (S)
	Rainfall	0.22 (S)		Rainfall	-0.17 (S)
	Relative Humidity	-0.45 (S)		Relative Humidity	0.00 (S)
	Wind speed	-0.46 (S)		Wind speed	0.06 (S)
Amaranthaceae	Mean Temperature	0.06 (P)	Amaranthaceae	Mean Temperature	-0.04 (P)
	Maximum Temperature	0.22 (P)		Maximum Temperature	-0.25 (P)
	Minimum Temperature	-0.28 (P)		Minimum Temperature	-0.06 (P)
				Rainfall	-0.44 (P)
	Relative Humidity	-0.50 (P)		Relative Humidity	0.09 (P)
	Wind speed	-0.26 (P)		Wind speed	-0.03 (P)
Urticaceae	Mean Temperature	-0.32 (S)	Urticaceae	Mean Temperature	-0.18 (S)
	Maximum Temperature	-0.25 (S)		Maximum Temperature	-0.09 (S)
	Minimum Temperature	-0.52 (S)		Minimum Temperature	-0.32 (S)
	Rainfall	-0.27 (S)		Rainfall	-0.17 (S)
	Relative Humidity	0.05 (S)		Relative Humidity	-0.37 (S)
	Wind speed	-0.59 (S)		Wind speed	0.44 (S)

Type of correlation; S: Spearman, P: Pearson.

Table S2.C2. Correlations between annual data by season of meteorological factors and the duration of pollen seasons. Summer and autumn were considered in Cupressaceae and Asteraceae. Only non-significant results are shown.

Summer (Jun-Jul-Aug)			Autumn (Sep-Oct-Nov)		
**Pollen type**	**Meteorological factor**	**Coefficient**	**Pollen type**	**Meteorological factor**	**Coefficient**
Cupressaceae	Mean Temperature	0.28 (P)	Cupressaceae	Mean Temperature	0.22 (S)
	Maximum Temperature	0.36 (P)		Maximum Temperature	0.19 (P)
	Minimum Temperature	0.34 (P)		Minimum Temperature	0.19 (P)
	Rainfall	0.33 (S)		Rainfall	-0.36 (P)
	Relative Humidity	-0.14 (P)		Relative Humidity	0.27 (P)
	Wind speed	-0.22 (P)		Wind speed	-0.04 (P)
Asteraceae	Mean Temperature	0.25 (P)	Asteraceae	Mean Temperature	0.38 (S)
	Maximum Temperature	0.23 (P)		Maximum Temperature	0.17 (P)
	Minimum Temperature	0.28 (P)		Minimum Temperature	0.55 (P)
	Rainfall	-0.18 (S)		Rainfall	-0.06 (P)
	Relative Humidity	0.03 (P)		Relative Humidity	0.37 (P)
	Wind speed	0.29 (P)		Wind speed	-0.26 (P)

Type of correlation; S: Spearman, P: Pearson.

Table S2.D1. Correlations between annual data by season of meteorological factors and the peak day of pollen seasons. Winter and spring were considered in Oleaceae, Pinaceae, Fagaceae, Poaceae, Amaranthaceae and Urticaceae. Only non-significant results are shown.

Winter (Dec-Jan-Feb)			Spring (Mar-Apr-May)		
**Pollen type**	**Meteorological factor**	**Coefficient**	**Pollen type**	**Meteorological factor**	**Coefficient**
Oleaceae	Mean Temperature	-0.07 (S)	Oleaceae		
	Maximum Temperature	-0.01 (S)			
	Minimum Temperature	-0.34 (S)			
	Rainfall	-0.42 (S)		Rainfall	0.13 (S)
	Relative Humidity	0.35 (S)		Relative Humidity	0.25 (S)
	Wind speed	-0.02 (S)		Wind speed	-0.10 (S)
Pinaceae	Mean Temperature	0.31 (P)	Pinaceae	Mean Temperature	-0.10 (P)
	Maximum Temperature	0.19 (P)		Maximum Temperature	-0.20 (P)
	Minimum Temperature	0.50 (P)		Minimum Temperature	0.12 (P)
	Rainfall	0.14 (S)		Rainfall	-0.05 (P)
	Relative Humidity	0.38 (P)		Relative Humidity	0.47 (P)
	Wind speed	0.59 (P)		Wind speed	-0.42 (P)
Fagaceae	Mean Temperature	0.21 (P)	Fagaceae	Mean Temperature	-0.41 (P)
	Maximum Temperature	0.44 (P)		Maximum Temperature	-0.61 (P)
	Minimum Temperature	-0.09 (P)		Minimum Temperature	-0.46 (P)
				Rainfall	-0.41 (P)
	Relative Humidity	-0.00 (P)		Relative Humidity	0.22 (P)
	Wind speed	0.07 (P)		Wind speed	0.02 (P)
Poaceae	Mean Temperature	0.05 (P)	Poaceae	Mean Temperature	-0.50 (P)
	Maximum Temperature	0.12 (P)		Maximum Temperature	-0.48 (P)
	Minimum Temperature	-0.05 (P)			
	Rainfall	-0.24 (S)		Rainfall	-0.09 (P)
	Relative Humidity	0.49 (P)		Relative Humidity	-0.16 (P)
	Wind speed	0.09 (P)		Wind speed	0.44 (P)
Amaranthaceae	Mean Temperature	0.08 (S)	Amaranthaceae	Mean Temperature	-0.05 (S)
	Maximum Temperature	0.31 (S)		Maximum Temperature	0.12 (S)
	Minimum Temperature	0.09 (S)		Minimum Temperature	0.05 (S)
	Rainfall	-0.30 (S)			
	Relative Humidity	-0.02 (S)		Relative Humidity	-0.25 (S)
	Wind speed	0.07 (S)		Wind speed	-0.61 (S)
Urticaceae	Mean Temperature	0.14 (P)	Urticaceae	Mean Temperature	-0.17 (P)
	Maximum Temperature	0.40 (P)		Maximum Temperature	-0.25 (P)
	Minimum Temperature	0.01 (P)		Minimum Temperature	-0.09 (P)
	Rainfall	-0.42 (S)		Rainfall	-0.13 (P)
	Relative Humidity	-0.14 (P)		Relative Humidity	0.41 (P)
	Wind speed	0.30 (P)		Wind speed	-0.47 (P)

Type of correlation; S: Spearman, P: Pearson.

Table S2.D2. Correlations between annual data by season of meteorological factors and the peak day of pollen seasons. Summer and autumn were considered in Cupressaceae and Asteraceae. Only non-significant results are shown.

Summer (Jun-Jul-Aug)			Autumn (Sep-Oct-Nov)		
**Pollen type**	**Meteorological factor**	**Coefficient**	**Pollen type**	**Meteorological factor**	**Coefficient**
Cupressaceae	Mean Temperature	0.11 (P)	Cupressaceae	Mean Temperature	-0.21 (S)
	Maximum Temperature	0.21 (P)		Maximum Temperature	-0.22 (P)
	Minimum Temperature	0.29 (P)		Minimum Temperature	-0.10 (P)
	Rainfall	-0.02 (S)		Rainfall	0.22 (P)
	Relative Humidity	0.18 (P)		Relative Humidity	0.32 (P)
	Wind speed	-0.34 (P)		Wind speed	-0.40 (P)
Asteraceae	Mean Temperature	0.56 (S)	Asteraceae	Mean Temperature	0.14 (S)
	Maximum Temperature	0.47 (S)		Maximum Temperature	0.31 (S)
	Minimum Temperature	0.50 (S)		Minimum Temperature	0.22 (S)
	Rainfall	-0.34 (S)		Rainfall	-0.08 (S)
	Relative Humidity	-0.36 (S)		Relative Humidity	0.22 (S)
	Wind speed	0.43 (S)		Wind speed	-0.08 (S)

Type of correlation; S: Spearman, P: Pearson.

Table S2.E1. Correlations between annual data by season of meteorological factors and the peak concentration of pollen seasons. Winter and spring were considered in Oleaceae, Pinaceae, Fagaceae, Poaceae, Amaranthaceae and Urticaceae. Only non-significant results are shown.

Winter (Dec-Jan-Feb)			Spring (Mar-Apr-May)		
**Pollen type**	**Meteorological factor**	**Coefficient**	**Pollen type**	**Meteorological factor**	**Coefficient**
Oleaceae	Mean Temperature	0.24 (P)	Oleaceae	Mean Temperature	0.12 (P)
	Maximum Temperature	0.19 (P)		Maximum Temperature	-0.35 (P)
	Minimum Temperature	0.06 (P)		Minimum Temperature	-0.00 (P)
	Rainfall	-0.22 (S)			
	Relative Humidity	0.27 (P)		Relative Humidity	0.15 (P)
	Wind speed	0.06 (P)		Wind speed	0.62 (P)
Pinaceae	Mean Temperature	0.07 (S)	Pinaceae	Mean Temperature	0.00 (S)
	Maximum Temperature	0.42 (S)		Maximum Temperature	0.02 (S)
	Minimum Temperature	0.04 (S)		Minimum Temperature	0.10 (S)
	Rainfall	-0.42 (S)		Rainfall	-0.43 (S)
	Relative Humidity	0.15 (S)		Relative Humidity	-0.21 (S)
	Wind speed	-0.22 (S)		Wind speed	-0.40 (S)
Fagaceae	Mean Temperature	-0.09 (P)	Fagaceae	Mean Temperature	0.30 (P)
	Maximum Temperature	-0.30 (P)		Maximum Temperature	0.26 (P)
	Minimum Temperature	-0.12 (P)		Minimum Temperature	0.24 (P)
	Rainfall	-0.07 (S)		Rainfall	-0.01 (P)
	Relative Humidity	-0.27 (P)		Relative Humidity	-0.28 (P)
	Wind speed	-0.16 (P)		Wind speed	0.41 (P)
Poaceae			Poaceae	Mean Temperature	0.07 (S)
				Maximum Temperature	-0.32 (S)
	Minimum Temperature	0.52 (S)		Minimum Temperature	0.21 (S)
	Rainfall	-0.59 (S)		Rainfall	-0.22 (S)
	Relative Humidity	-0.13 (S)		Relative Humidity	0.31 (S)
	Wind speed	0.26 (S)		Wind speed	-0.39 (S)
Amaranthaceae	Mean Temperature	0.50 (P)	Amaranthaceae	Mean Temperature	0.35 (P)
	Maximum Temperature	0.46 (P)		Maximum Temperature	0.32 (P)
				Minimum Temperature	0.19 (P)
	Rainfall	0.07 (S)		Rainfall	0.15 (P)
	Relative Humidity	0.54 (P)		Relative Humidity	-0.47 (P)
	Wind speed	0.29 (P)		Wind speed	0.06 (P)
Urticaceae	Mean Temperature	0.38 (P)	Urticaceae	Mean Temperature	0.23 (P)
	Maximum Temperature	0.22 (P)		Maximum Temperature	-0.32 (P)
	Minimum Temperature	0.06 (P)		Minimum Temperature	0.17 (P)
	Rainfall	-0.22 (S)		Rainfall	0.52 (P)
	Relative Humidity	-0.47 (P)			
	Wind speed	0.18 (P)		Wind speed	0.15 (P)

Type of correlation; S: Spearman, P: Pearson.

Table S2.E2. Correlations between annual data by season of meteorological factors and the peak concentration of pollen seasons. Summer and autumn were considered in Cupressaceae and Asteraceae. Only non-significant results are shown.

Summer (Jun-Jul-Aug)			Autumn (Sep-Oct-Nov)		
**Pollen type**	**Meteorological factor**	**Coefficient**	**Pollen type**	**Meteorological factor**	**Coefficient**
Cupressaceae	Mean Temperature	-0.28 (P)	Cupressaceae	Mean Temperature	-0.47 (S)
	Maximum Temperature	-0.30 (P)		Maximum Temperature	-0.25 (P)
	Minimum Temperature	-0.37 (P)		Minimum Temperature	-0.57 (P)
	Rainfall	0.16 (S)		Rainfall	0.09 (P)
	Relative Humidity	-0.15 (P)		Relative Humidity	-0.48 (P)
	Wind speed	0.03 (P)		Wind speed	0.18 (P)
Asteraceae	Mean Temperature	-0.41 (P)	Asteraceae	Mean Temperature	-0.38 (S)
	Maximum Temperature	-0.32 (P)		Maximum Temperature	-0.30 (P)
	Minimum Temperature	-0.43 (P)		Minimum Temperature	-0.63 (P)
	Rainfall	0.04 (S)		Rainfall	0.0 (P)
	Relative Humidity	-0.27 (P)			
	Wind speed	0.11 (P)		Wind speed	-0.02 (P)

Type of correlation; S: Spearman, P: Pearson.

Table S3.A. Correlations between annual data by period 1 of meteorological factors and the start of pollen seasons. Only non-significant results are shown.

Pollen type	Meteorological factor	Coefficient	Pollen type	Meteorological factor	Coefficient
Oleaceae	Mean Temperature	0.11 (P)	Poaceae		
	Maximum Temperature	-0.03 (P)		Maximum Temperature	0.62 (P)
	Minimum Temperature	0.15 (P)			
	Rainfall	0.29 (P)		Rainfall	-0.15 (P)
				Relative Humidity	-0.18 (P)
	Wind speed	0.42 (P)		Wind speed	0.49 (S)
Cupressaceae			Amaranthaceae	Mean Temperature	0.24 (P)
				Maximum Temperature	0.51 (P)
				Minimum Temperature	0.33 (P)
	Rainfall	0.44 (P)		Rainfall	0.01 (P)
	Relative Humidity	-0.56 (P)		Relative Humidity	0.09 (P)
	Wind speed	0.11 (P)		Wind speed	-0.22 (P)
Pinaceae	Mean Temperature	-0.58 (S)	Asteraceae		
	Maximum Temperature	-0.35 (S)			
	Minimum Temperature	-0.46 (S)			
	Rainfall	0.18 (S)			
	Relative Humidity	0.37 (S)		Relative Humidity	-0.18 (S)
	Wind speed	-0.48 (S)		Wind speed	0.14 (S)
Fagaceae	Mean Temperature	0.47 (P)	Urticaceae	Mean Temperature	0.33 (S)
	Maximum Temperature	0.59 (P)		Maximum Temperature	0.47 (S)
	Minimum Temperature	0.28 (S)		Minimum Temperature	0.40 (S)
	Rainfall	-0.53 (P)		Rainfall	0.04 (S)
	Relative Humidity	0.29 (P)		Relative Humidity	0.52 (S)
	Wind speed	0.05 (S)		Wind speed	-0.24 (S)

Period 1; from 28 days previous to the start of pollen season to the day before the start of pollen season.
Period 2; from the day that the pollen season starts to the day before the peak day into the pollen season.
Period 3; from the peak day to the day that pollen season ends.
Period 4; the sum of period 1, 2 and 3.
Type of correlation; S: Spearman, P: Pearson.

Table S3.B. Correlations between annual data by period 2 of meteorological factors and the peak day of pollen seasons. Only non-significant results are shown.

Pollen type	Meteorological factor	Coefficient	Pollen type	Meteorological factor	Coefficient
Oleaceae	Mean Temperature	0.51 (S)	Poaceae	Mean Temperature	-0.00 (P)
				Maximum Temperature	-0.05 (P)
	Minimum Temperature	0.25 (S)		Minimum Temperature	-0.03 (P)
	Rainfall	0.04 (S)		Rainfall	0.50 (S)
	Relative Humidity	-0.46 (S)		Relative Humidity	-0.10 (S)
	Wind speed	0.18 (S)		Wind speed	0.25 (S)
Cupressaceae	Mean Temperature	0.28 (P)	Amaranthaceae		
	Maximum Temperature	0.18 (P)			
	Minimum Temperature	0.25 (P)			
	Rainfall	0.10 (P)		Rainfall	-0.04 (S)
	Relative Humidity	0.10 (P)		Relative Humidity	0.02 (S)
	Wind speed	0.35 (P)		Wind speed	-0.09 (S)
Pinaceae			Asteraceae	Mean Temperature	0.25 (S)
				Maximum Temperature	0.28 (S)
				Minimum Temperature	0.21 (S)
	Rainfall	0.16 (S)		Rainfall	0.45 (S)
	Relative Humidity	-0.09 (S)		Relative Humidity	-0.02 (S)
	Wind speed	-0.05 (P)		Wind speed	0.31 (S)
Fagaceae	Mean Temperature	0.53 (P)	Urticaceae		
	Maximum Temperature	0.41 (P)			
	Minimum Temperature	0.48 (P)			
	Rainfall	0.25 (S)		Rainfall	-0.37 (P)
	Relative Humidity	-0.12 (P)		Relative Humidity	0.22 (P)
	Wind speed	0.66 (P)		Wind speed	-0.41 (S)

Period 1; from 28 days previous to the start of pollen season to the day before the start of pollen season.
Period 2; from the day that the pollen season starts to the day before the peak day into the pollen season.
Period 3; from the peak day to the day that pollen season ends.
Period 4; the sum of period 1, 2 and 3.
Type of correlation; S: Spearman, P: Pearson.

Table S3.C. Correlations between annual data by period 2 of meteorological factors and the peak concentration of pollen seasons. Only non-significant results are shown.

Pollen type	Meteorological factor	Coefficient	Pollen type	Meteorological factor	Coefficient
Oleaceae	Mean Temperature	0.41 (P)	Poaceae	Mean Temperature	-0.15 (S)
	Maximum Temperature	-0.29 (P)		Maximum Temperature	-0.38 (S)
	Minimum Temperature	0.43 (P)		Minimum Temperature	-0.16 (S)
	Rainfall	0.24 (S)		Rainfall	0.35 (S)
	Relative Humidity	0.07 (P)		Relative Humidity	0.48 (S)
	Wind speed	0.19 (P)			
Cupressaceae	Mean Temperature	-0.65 (P)	Amaranthaceae	Mean Temperature	-0.12 (S)
	Maximum Temperature	-0.49 (P)		Maximum Temperature	-0.23 (P)
	Minimum Temperature	-0.64 (P)		Minimum Temperature	-0.31 (S)
	Rainfall	-0.33 (P)		Rainfall	0.15 (S)
	Relative Humidity	-0.20 (P)		Relative Humidity	-0.46 (P)
				Wind speed	-0.17 (P)
Pinaceae	Mean Temperature	-0.06 (S)	Asteraceae		
	Maximum Temperature	-0.02 (S)			
	Minimum Temperature	-0.21 (S)		Minimum Temperature	-0.59 (S)
				Rainfall	-0.26 (S)
	Relative Humidity	-0.21 (S)		Relative Humidity	-0.43 (P)
	Wind speed	-0.32 (S)		Wind speed	0.57 (P)
Fagaceae	Mean Temperature	-0.27 (P)	Urticaceae	Mean Temperature	0.40 (S)
	Maximum Temperature	-0.23 (P)		Maximum Temperature	-0.08 (P)
	Minimum Temperature	-0.34 (P)		Minimum Temperature	0.10 (S)
	Rainfall	-0.02 (S)		Rainfall	0.57 (P)
	Relative Humidity	-0.22 (P)		Relative Humidity	0.45 (P)
	Wind speed	-0.18 (P)		Wind speed	0.12 (S)

Period 1; from 28 days previous to the start of pollen season to the day before the start of pollen season.
Period 2; from the day that the pollen season starts to the day before the peak day into the pollen season.
Period 3; from the peak day to the day that pollen season ends.
Period 4; the sum of period 1, 2 and 3.
Type of correlation; S: Spearman, P: Pearson.

Table S3.D. Correlations between annual data by period 3 of meteorological factors and the end of pollen seasons. Only non-significant results are shown.

Pollen type	Meteorological factor	Coefficient	Pollen type	Meteorological factor	Coefficient
Oleaceae	Mean Temperature	-0.02 (S)	Poaceae	Mean Temperature	0.14 (S)
	Maximum Temperature	-0.06 (S)		Maximum Temperature	0.33 (S)
	Minimum Temperature	0.22 (S)		Minimum Temperature	0.22 (S)
	Rainfall	0.36 (S)		Rainfall	0.44 (S)
				Relative Humidity	0.43 (S)
	Wind speed	-0.43 (S)		Wind speed	-0.42 (S)
Cupressaceae	Mean Temperature	0.62 (P)	Amaranthaceae	Mean Temperature	0.05 (S)
				Maximum Temperature	-0.44 (S)
	Minimum Temperature	0.53 (P)		Minimum Temperature	0.04 (S)
	Rainfall	-0.33 (P)			
	Relative Humidity	0.08 (P)		Relative Humidity	0.13 (S)
	Wind speed	-0.44 (P)		Wind speed	-0.21 (S)
Pinaceae	Mean Temperature	0.38 (P)	Asteraceae	Mean Temperature	0.27 (P)
	Maximum Temperature	0.43 (P)		Maximum Temperature	0.23 (P)
	Minimum Temperature	0.39 (P)		Minimum Temperature	0.51 (P)
	Rainfall	-0.53 (S)		Rainfall	-0.02 (S)
	Relative Humidity	-0.20 (S)		Relative Humidity	0.15 (P)
	Wind speed	-0.06 (P)		Wind speed	0.52 (P)
Fagaceae	Mean Temperature	0.27 (P)	Urticaceae	Mean Temperature	0.30 (S)
	Maximum Temperature	0.26 (P)		Maximum Temperature	0.18 (S)
	Minimum Temperature	0.34 (P)		Minimum Temperature	0.32 (S)
	Rainfall	-0.10 (S)		Rainfall	0.28 (S)
	Relative Humidity	0.55 (P)		Relative Humidity	0.52 (S)
	Wind speed	-0.57 (P)		Wind speed	-0.16 (S)

Period 1; from 28 days previous to the start of pollen season to the day before the start of pollen season.
Period 2; from the day that the pollen season starts to the day before the peak day into the pollen season.
Period 3; from the peak day to the day that pollen season ends.
Period 4; the sum of period 1, 2 and 3.
Type of correlation; S: Spearman, P: Pearson.

Table S3.E. Correlations between annual data by period 4 of meteorological factors and the duration of pollen seasons. Only non-significant results are shown.

Pollen type	Meteorological factor	Coefficient	Pollen type	Meteorological factor	Coefficient
Oleaceae	Mean Temperature	0.15 (S)	Poaceae	Mean Temperature	0.24 (S)
	Maximum Temperature	0.25 (S)		Maximum Temperature	0.36 (S)
	Minimum Temperature	0.34 (S)		Minimum Temperature	0.22 (S)
	Rainfall	-0.30 (S)		Rainfall	-0.20 (S)
	Relative Humidity	-0.27 (S)		Relative Humidity	0.13 (S)
	Wind speed	-0.24 (S)		Wind speed	-0.57 (S)
Cupressaceae			Amaranthaceae	Mean Temperature	-0.46 (P)
				Maximum Temperature	-0.63 (P)
				Minimum Temperature	-0.45 (P)
	Rainfall	-0.28 (P)		Rainfall	0.27 (P)
	Relative Humidity	-0.17 (P)		Relative Humidity	0.47 (P)
	Wind speed	-0.56 (S)		Wind speed	-0.09 (P)
Pinaceae	Mean Temperature	0.25 (P)	Asteraceae		
	Maximum Temperature	-0.12 (P)			
	Minimum Temperature	0.16 (P)			
	Rainfall	-0.30 (P)		Rainfall	0.15 (P)
	Relative Humidity	-0.07 (P)		Relative Humidity	0.14 (P)
	Wind speed	0.17 (P)		Wind speed	0.18 (P)
Fagaceae	Mean Temperature	0.05 (P)	Urticaceae	Mean Temperature	-0.01 (S)
	Maximum Temperature	-0.07 (P)		Maximum Temperature	-0.16 (S)
	Minimum Temperature	0.02 (P)		Minimum Temperature	0.24 (S)
	Rainfall	0.31 (P)		Rainfall	0.26 (S)
	Relative Humidity	0.13 (P)			
	Wind speed	-0.02 (P)		Wind speed	-0.17 (S)

Period 1; from 28 days previous to the start of pollen season to the day before the start of pollen season.
Period 2; from the day that the pollen season starts to the day before the peak day into the pollen season.
Period 3; from the peak day to the day that pollen season ends.
Period 4; the sum of period 1, 2 and 3.
Type of correlation; S: Spearman, P: Pearson.
