# Supplementary material for: Aerobiological and clinical study in the semidesertic area of the Southeastern of Spain
Source: Front Allergy. 2024 Mar 25;5:1328940. doi: 10.3389/falgy.2024.1328940 (PMC10999673; doi:10.3389/falgy.2024.1328940)
Supplement: Supplementary file 2 [file Datasheet2.pdf]

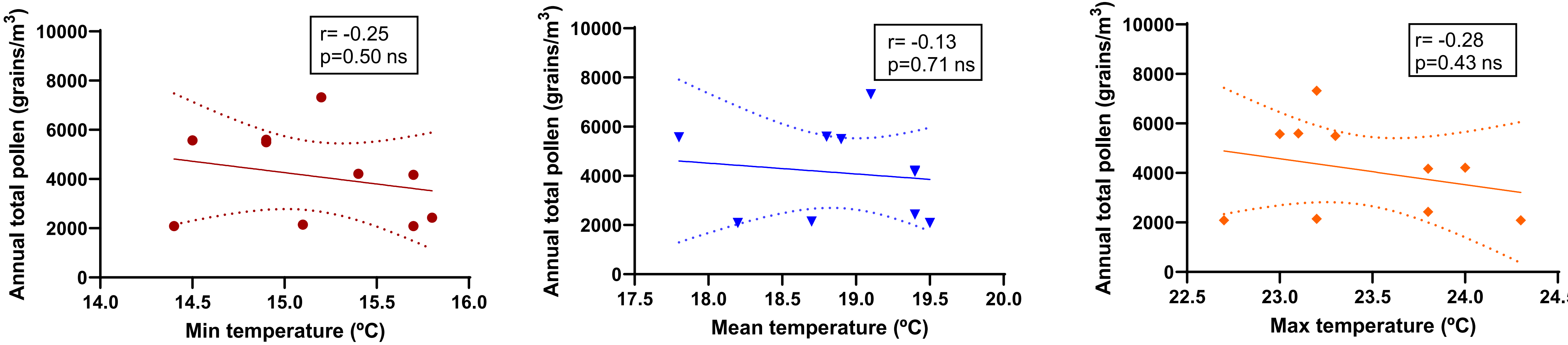

Figure S1.A. Correlations between Oleaceae annual total pollen amount and annual mean, maximum and minimum temperatures. Pearson´s r and p-value are shown.

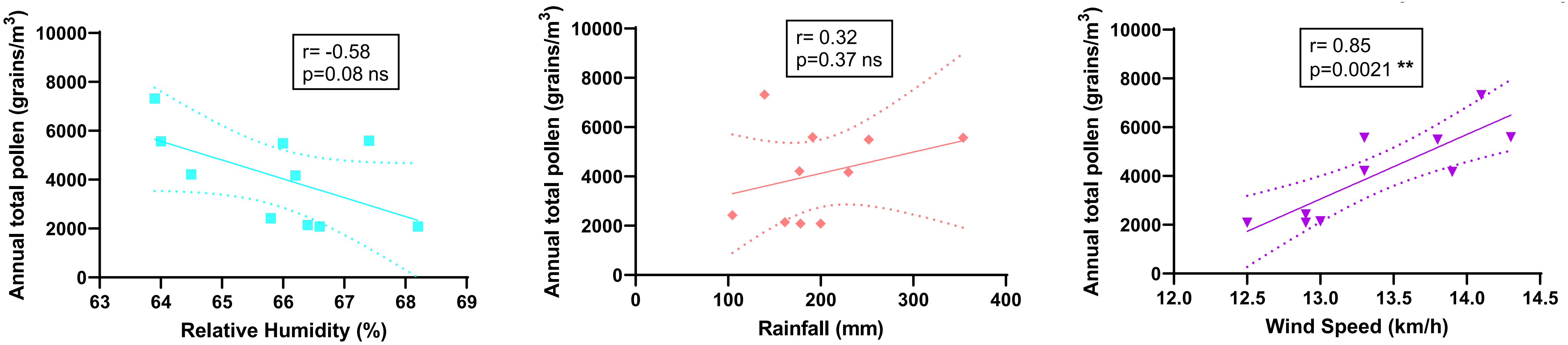

Figure S1.B. Correlations between Oleaceae annual total pollen amount and annual relative humidity, rainfall and wind speed. Pearson´s r and p-value are shown.

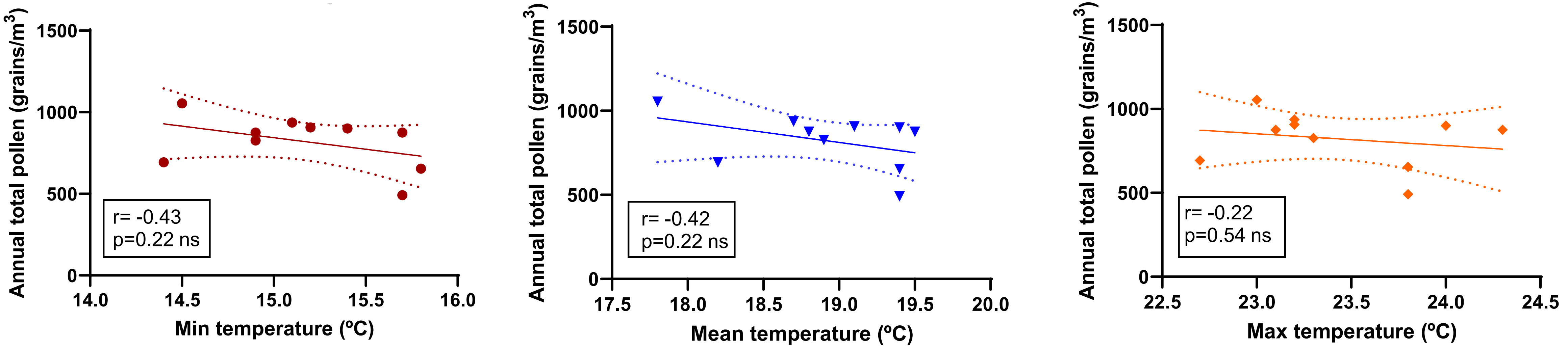

Figure S2.B. Correlations between Amaranthaceae annual total pollen amount and annual mean, maximum and minimum temperatures. Pearson´s r and p-value are shown.

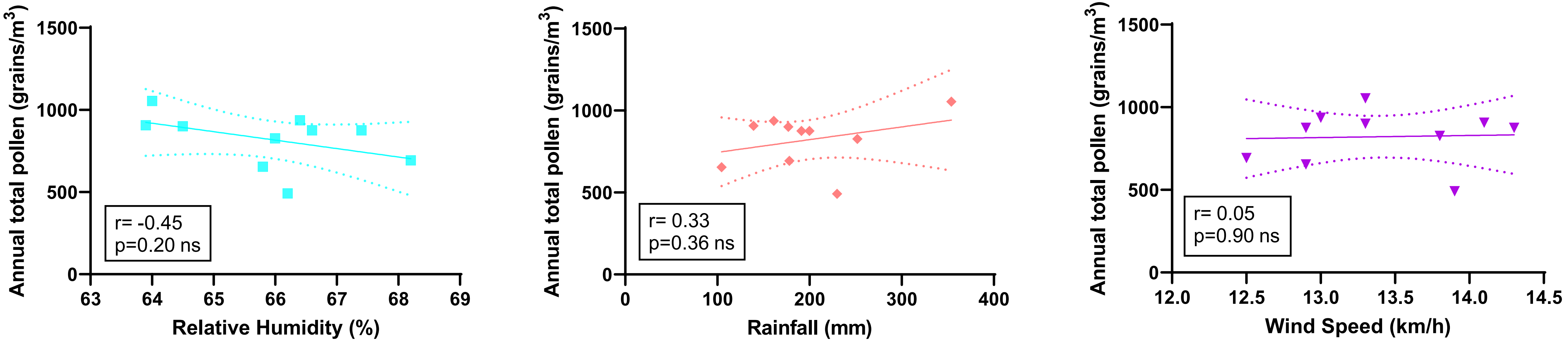

Figure S2.B. Correlations between Amaranthaceae annual total pollen amount and annual relative humidity, rainfall and wind speed. Pearson´s r and p-value are shown.

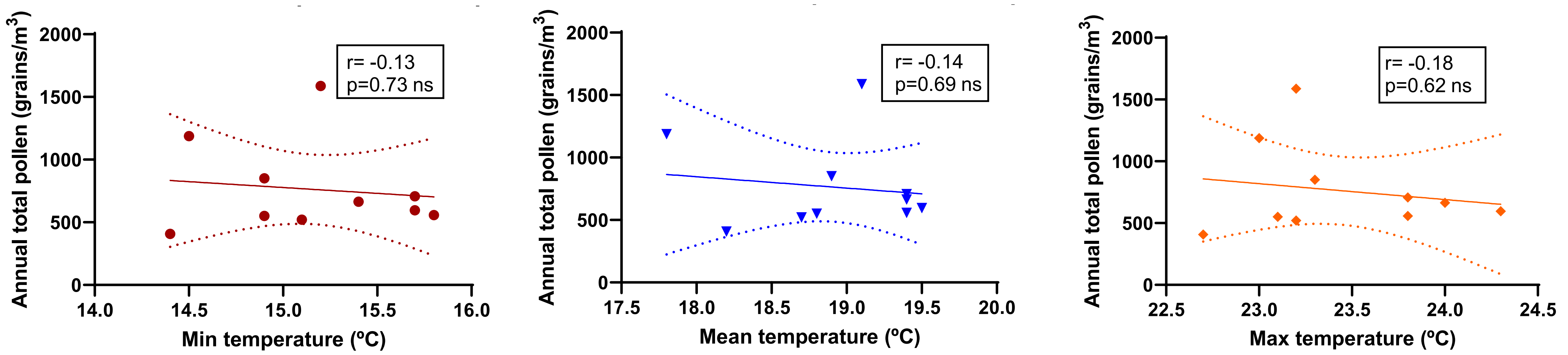

Figure S3.A. Correlations between Poaceae annual total pollen amount and annual mean, maximum and minimum temperatures. Pearson´s r and p-value are shown.

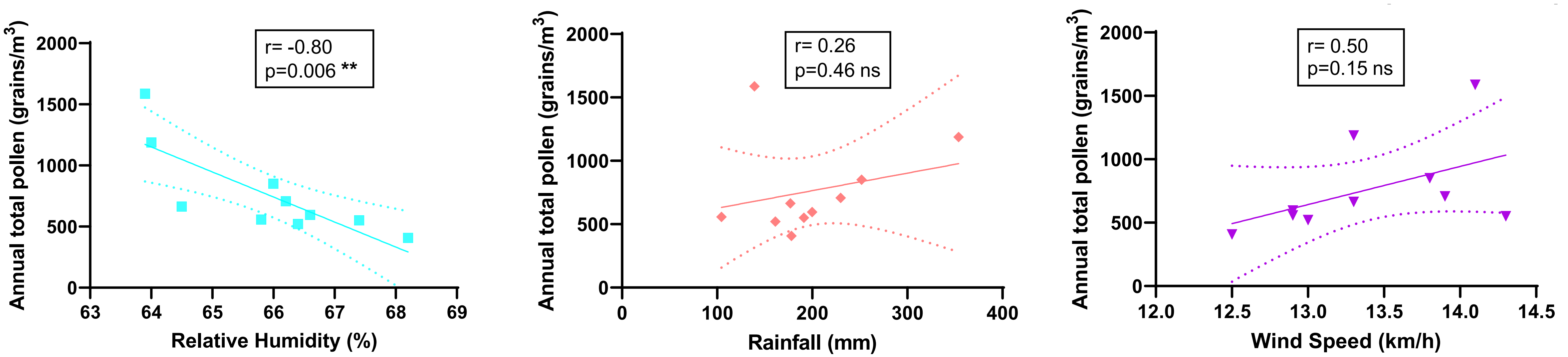

Figure S3.B. Correlations between Poaceae annual total pollen amount and annual relative humidity, rainfall and wind speed. Pearson´s r and p-value are shown.

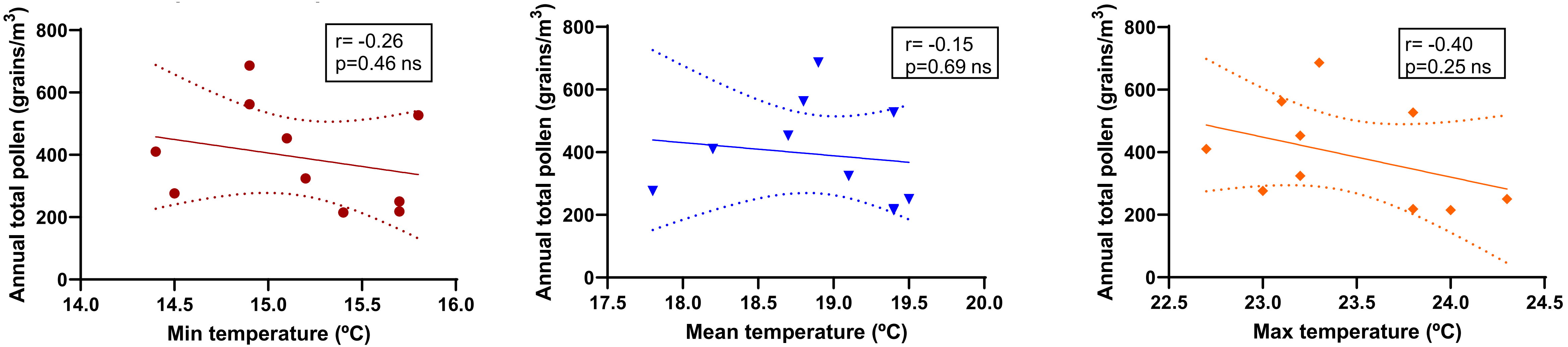

Figure S4.A. Correlations between Cupressaceae annual total pollen amount and annual mean, maximum and minimum temperatures. Pearson´s r and p-value and are shown.

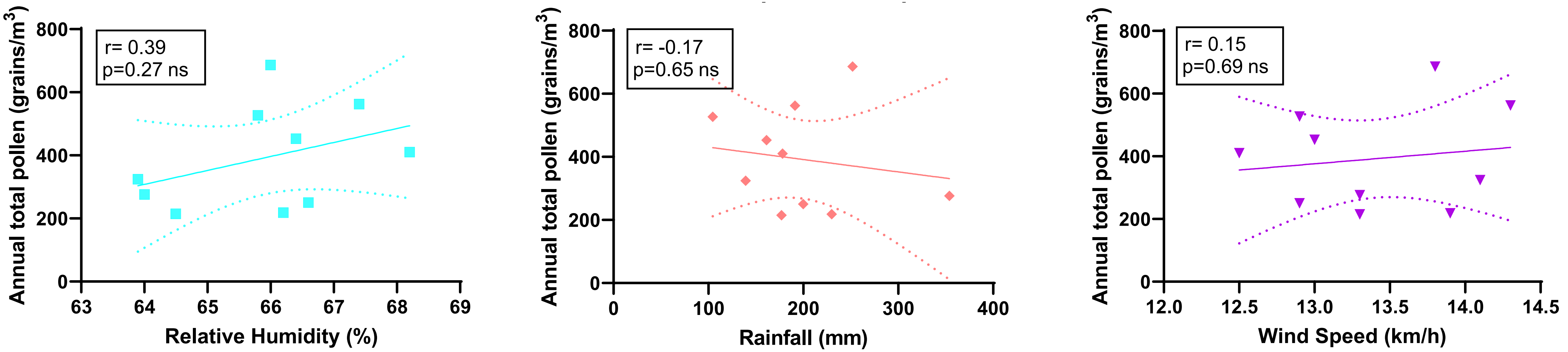

Figure S4.B. Correlations between Cupressaceae annual total pollen amount and annual relative humidity, rainfall and wind speed. Pearson´s r and p-value are shown.

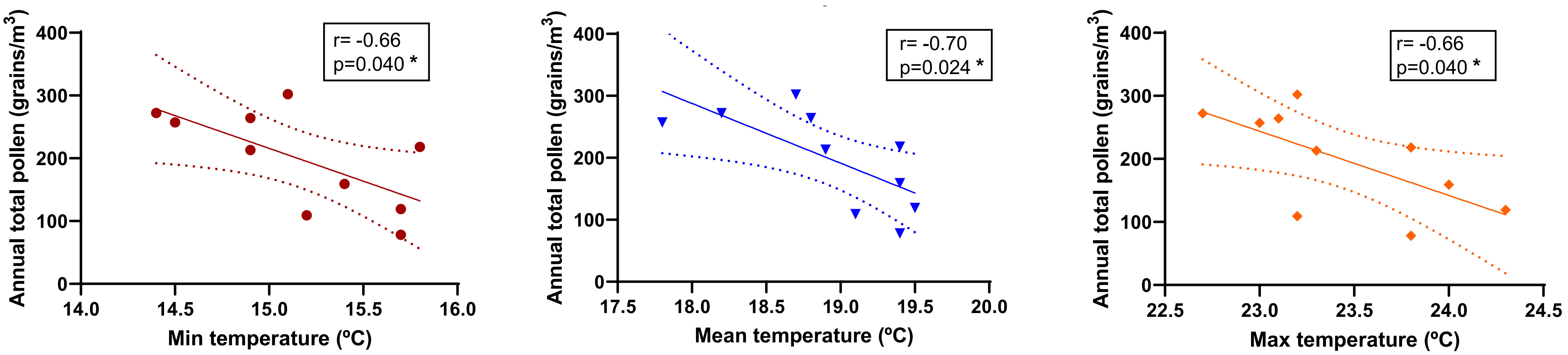

Figure S5.A. Correlations between Asteraceae annual total pollen amount and annual mean, maximum and minimum temperatures. Pearson´s r and p-value and are shown.

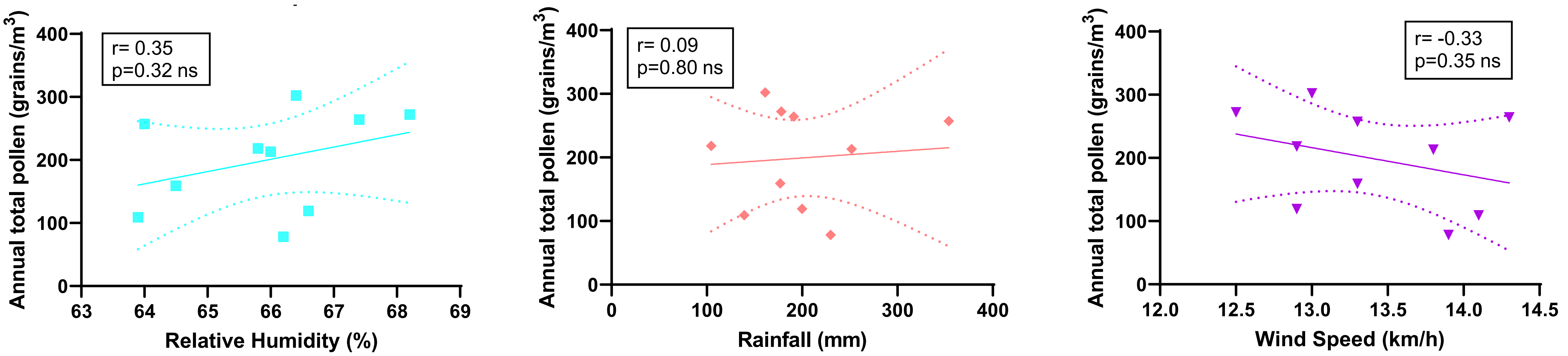

Figure S5.B. Correlations between Asteraceae annual total pollen amount and annual relative humidity, rainfall and wind speed. Pearson´s r and p-value are shown.

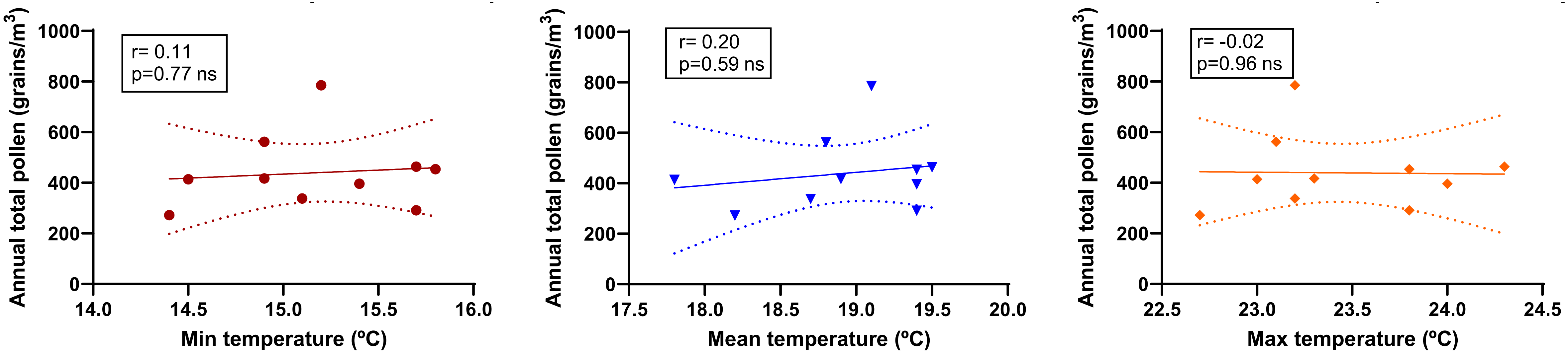

Figure S6.A. Correlations between Urticaceae annual total pollen amount and annual mean, maximum and minimum temperatures. Pearson´s r and p-value and are shown.

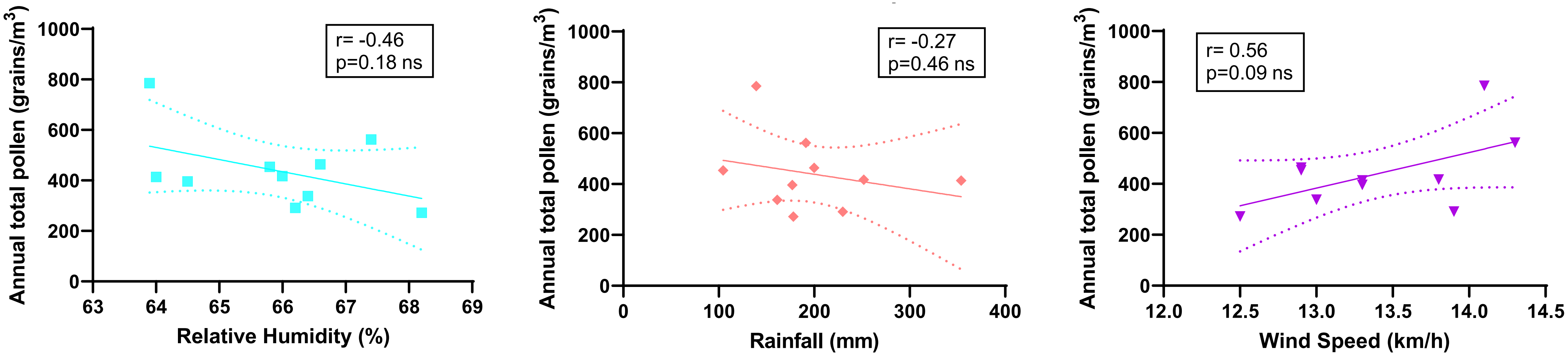

Figure S6.B. Correlations between Urticaceae annual total pollen amount and annual relative humidity, rainfall and wind speed. Pearson´s r and p-value are shown.

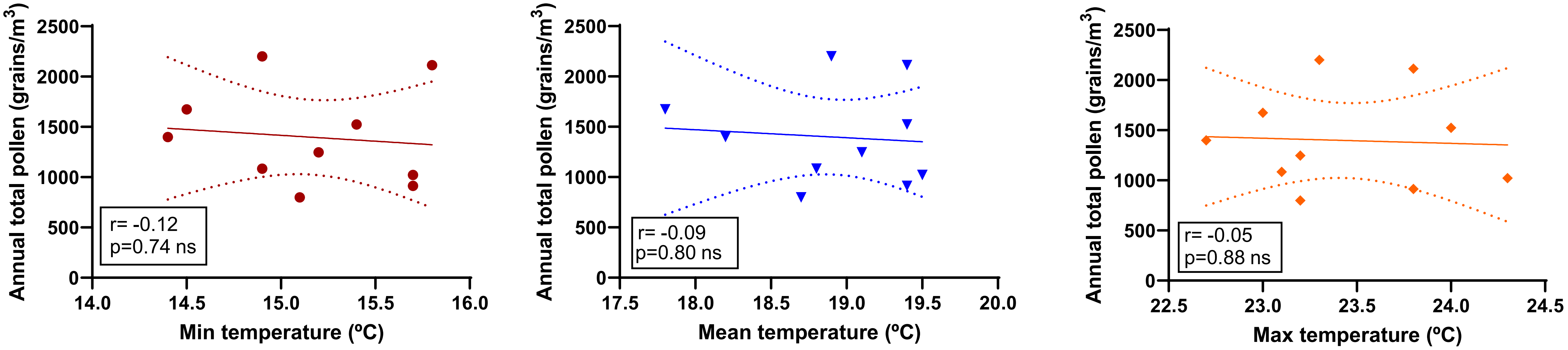

Figure S7.A. Correlations between Fagaceae annual total pollen amount and annual mean, maximum and minimum temperatures. Pearson´s r and p-value and are shown.

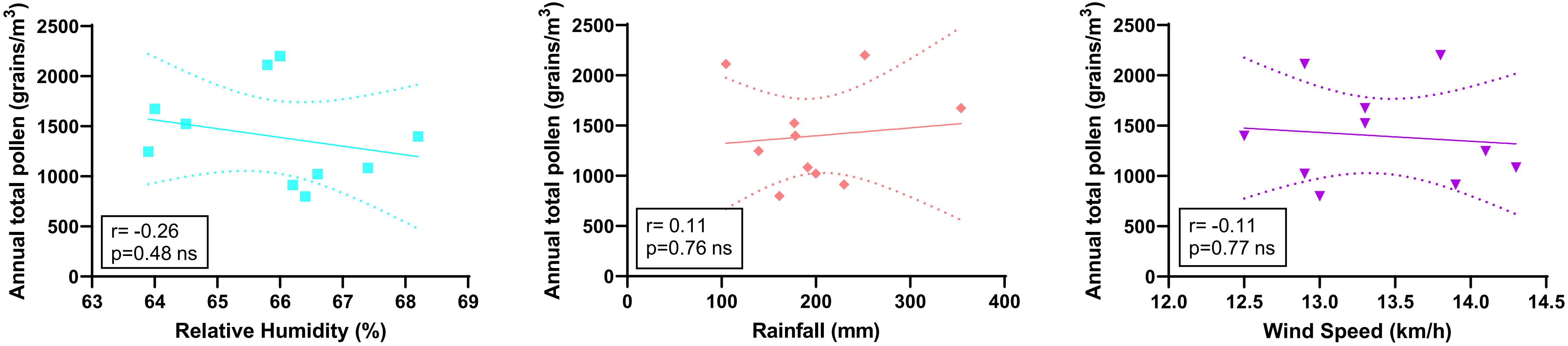

Figure S7.B. Correlations between Fagaceae annual total pollen amount and annual relative humidity, rainfall and wind speed. Pearson´s r and p-value are shown.

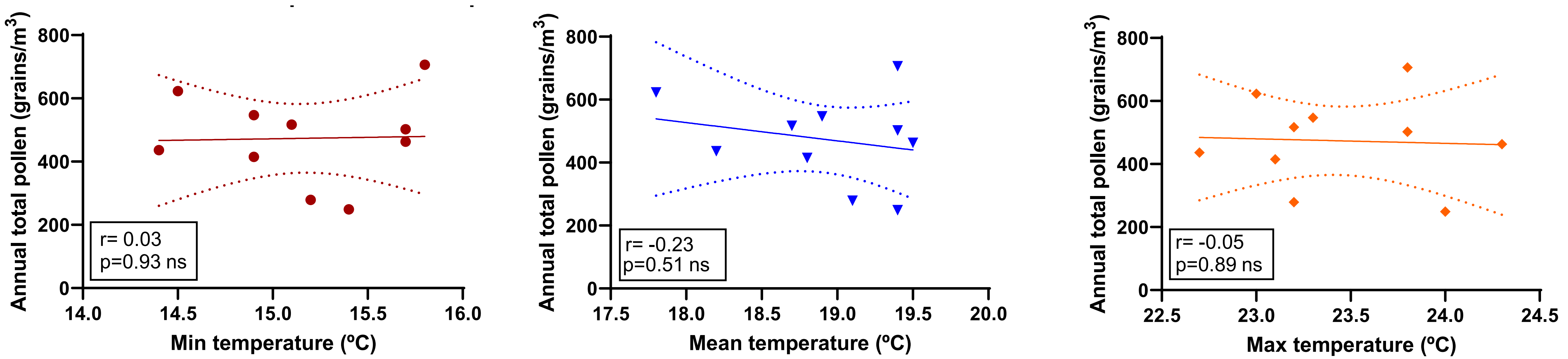

Figure S8.A. Correlations between Pinaceae annual total pollen amount and annual mean, maximum and minimum temperatures. Pearson´s r and p-value and are shown.

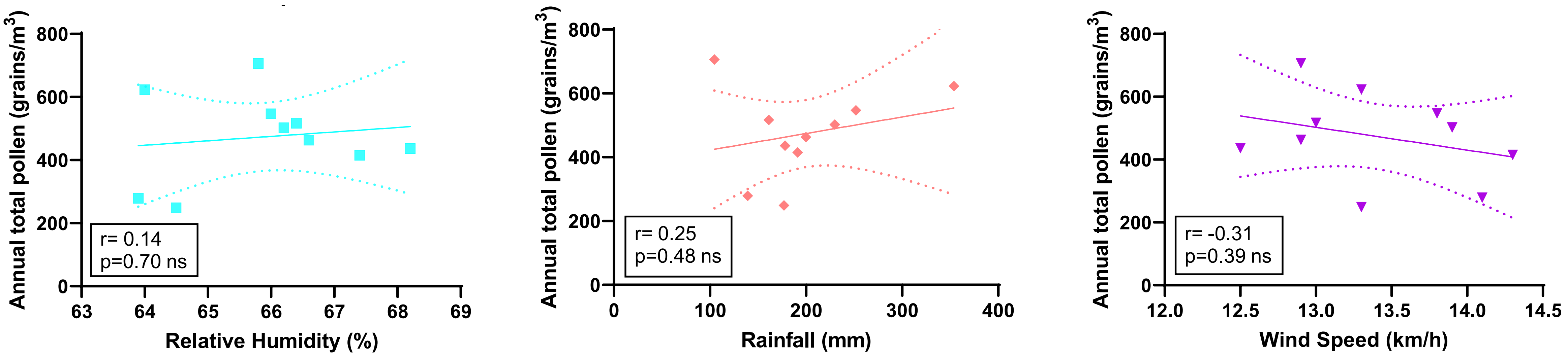

Figure S8.B. Correlations between Pinaceae annual total pollen amount and annual relative humidity, rainfall and wind speed. Pearson´s r and p-value are shown.
